# An archaeal virus-encoded anti-CRISPR protein inhibits type III-B immunity by inhibiting Cas RNP complex turnover

**DOI:** 10.1093/nar/gkad804

**Published:** 2023-10-18

**Authors:** Jilin Liu, Qian Li, Xiaojie Wang, Zhenzhen Liu, Qing Ye, Tao Liu, Saifu Pan, Nan Peng

**Affiliations:** National Key Laboratory of Agricultural Microbiology, Hubei Hongshan Laboratory, College of Life Science and Technology, Huazhong Agricultural University, Wuhan 430070, P.R. China; National Key Laboratory of Agricultural Microbiology, Hubei Hongshan Laboratory, College of Life Science and Technology, Huazhong Agricultural University, Wuhan 430070, P.R. China; National Key Laboratory of Agricultural Microbiology, Hubei Hongshan Laboratory, College of Life Science and Technology, Huazhong Agricultural University, Wuhan 430070, P.R. China; Antibiotics Research and Re-evaluation Key Laboratory of Sichuan Province, Sichuan Industrial Institute of Antibiotics, School of Pharmacy, Chengdu University, 610106, Chengdu, P. R. China; National Key Laboratory of Agricultural Microbiology, Hubei Hongshan Laboratory, College of Life Science and Technology, Huazhong Agricultural University, Wuhan 430070, P.R. China; National Key Laboratory of Agricultural Microbiology, Hubei Hongshan Laboratory, College of Life Science and Technology, Huazhong Agricultural University, Wuhan 430070, P.R. China; National Key Laboratory of Agricultural Microbiology, Hubei Hongshan Laboratory, College of Life Science and Technology, Huazhong Agricultural University, Wuhan 430070, P.R. China; National Key Laboratory of Agricultural Microbiology, Hubei Hongshan Laboratory, College of Life Science and Technology, Huazhong Agricultural University, Wuhan 430070, P.R. China

## Abstract

CRISPR–Cas systems are widespread in prokaryotes and provide adaptive immune against viral infection. Viruses encode a type of proteins called anti-CRISPR to evade the immunity. Here, we identify an archaeal virus-encoded anti-CRISPR protein, AcrIIIB2, that inhibits Type III-B immunity. We find that AcrIIIB2 inhibits Type III-B CRISPR–Cas immunity *in vivo* regardless of viral early or middle-/late-expressed genes to be targeted. We also demonstrate that AcrIIIB2 interacts with Cmr4α subunit, forming a complex with target RNA and Cmr-α ribonucleoprotein complex (RNP). Furtherly, we discover that AcrIIIB2 inhibits the RNase activity, ssDNase activity and cOA synthesis activity of Cmr-α RNP *in vitro* under a higher target RNA-to-Cmr-α RNP ratio and has no effect on Cmr-α activities at the target RNA-to-Cmr-α RNP ratio of 1. Our results suggest that once the target RNA is cleaved by Cmr-α RNP, AcrIIIB2 probably inhibits the disassociation of cleaved target RNA, therefore blocking the access of other target RNA substrates. Together, our findings highlight the multiple functions of a novel anti-CRISPR protein on inhibition of the most complicated CRISPR–Cas system targeting the genes involved in the whole life cycle of viruses.

## Graphical Abstract

**Graphical Abstract:**
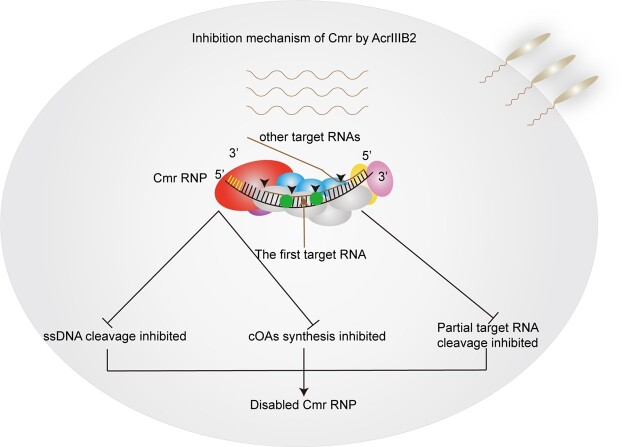

## Introduction

Bacteria and archaea have evolved multiple immune systems including the well-studied CRISPR–Cas systems (1,2) to prevent the invasion of mobile genetic elements (MGEs) (3). CRISPR–Cas systems provide adaptative immunity by memorizing invading nucleic acids of phages (4) and plasmids (5). These systems are divided into two classes, six types and 33 subtypes (1), which share similar immunity stages: stage 1, the Cas1–Cas2 complex integrates the fragments of foreign genetic elements into CRISPR arrays to form new spacers; stage 2, CRISPR arrays are transcribed and processed into mature crRNAs by Cas proteins or cellular ribonuclease and stage 3, mature crRNAs guide the interference complex to cleave invading nucleic acids (6). Among the six types, Type III CRISPR–Cas systems are the most complex and intricate immune systems described thus far (1,7–19). These systems share a unique target RNA-dependent mechanism, and the well-studied Type III-A (also known as Csm) and Type III-B (Cmr) systems have multiple functions including specific RNA cleavage, cOA (cyclic oligoadenylates) synthesis and non-specific single-stranded DNA (ssDNA) cleavage. Recently reported Type III-E systems binds a crRNA and the putative protease Csx29 to cleavage the accessory protein Csx30 (8–13,15–18). Besides, Type III CRISPR–Cas-associated RNase Csm6/Csx1 non-specifically degrades RNA (20,21) or the associated protease CalpL proteolytic release of a σ factor to initiate the transcription of genes probably for antiviral function (19), when they are activated by the cOAs (19–21).

MGEs have evolved diverse strategies to defend against these antiviral systems. Among them, point mutations at CRISPR–Cas targeted sites (22) and the formation of nucleus-like structures (23,24) are effective strategies to prevent target recognition or cleavage. MGEs also encode a variety of small proteins, termed anti-CRISPR proteins (Acrs), to inactivate the CRISPR–Cas systems (25,26). Currently, ∼90 families of Acrs have been reported to fight against 13 subtypes of CRISPR–Cas systems (25).

Two Acr families (AcrIII1 and AcrIIIB1) have been identified to defend against Type III CRISPR–Cas systems (27,28). AcrIII1 is a new family of ring nuclease encoded by archaea virus SIRV1 and other species including archaea virus and bacteriophages, and AcrIII1 degrades cyclic tetra-adenylate (cA4) that acts as the second messenger to activate non-specific RNase Csx1 (27). AcrIIIB1 is encoded by SIRV2, almost all members of Rudiviridae, and more than half of the Lipothrixviridae family members, and AcrIIIB1 specifically inhibits Type III-B immunity from targeting viral middle/late-expressed genes by directly binding Cmr effector complexes (28). Both of these Acrs suppress Type III CRISPR–Cas systems from targeting the middle/late-expressed viral genes (27,28).

Here, we identified a *Sulfolobus* virus SIRV3-encoded anti-CRISPR protein and designated it as AcrIIIB2, which is the first Acr identified to inhibit Type III-B immunity targeting both viral early-expressed and middle-/late-expressed genes. We revealed that AcrIIIB2 interacts with Cmr4α subunit to form a complex with target RNA and Cmr-α RNP, preventing dissociation of target RNA from the Cmr-α complex, thereby shutting down the turnover of the complex to inactivate RNase, ssDNase and cOA synthesis activities of Cmr-α RNP.

## Materials and methods

### Construction of *Escherichia coli* expression plasmids and strains

To express the proteins in *E. coli*, the coding sequence of AcrIIIB2 (*BHS13_gp40*) was amplified from the genomic DNA of SIRV3 (*Sulfolobus islandicus* Rod-Shaped Virus 3) by PCR using the primers listed in Supplementary Table S3. The gene fragments were inserted into pET30a between the Nde I and Xho I restriction sites, such that the encoded proteins have a C-terminal 6 × His tag. And the gene fragments were also inserted into pGEX-6p-1 between the BamH I and Sal I restriction sites to generate an N-terminal GST-tagged AcrIIIB2. The gene fragments were inserted into pET28a-SUMO between BamH I and Hind III restriction sites to generate and N-terminal 6× His-SUMO-tagged AcrIIIB2. The gene fragments were inserted into pMAL-C5X-His between Nde I and Hind III restriction sites to generate an N-terminal MBP-TEV-tagged AcrIIIB2. The gene fragments were inserted into pACYC_Duet-1 between Nco I and BamH I restriction sites to generate non-tagged AcrIIIB2. The coding sequences of Cmr2α (*SIRE_RS04505*), Cmr4α (*SIRE_RS04485*), Cmr5α (*SIRE_RS04490*) and Csx1 (*SIRE_RS04455*) were amplified from the genomic DNA of *S. islandicus* REY15A by PCR using the primers listed in Supplementary Table S3. The *cmr4α* gene fragment was inserted into pET30a between the Nde I and Sal I restriction sites to generate C-terminal 6 × His-tagged Cmr4α. The *cmr4α* gene fragment was inserted into pACYCDuet-1 between the Nde I and Xho I restriction sites to generate C-terminal 6× His-tagged Cmr4α. The *cmr5α* and *csx1* gene fragments were inserted into pET30a between the Nde I and Xho I restriction sites. The *cmr5α* gene fragment was inserted into pACYC_Duet-1 between the Nde I and Xho I restriction sites to generate C-terminal 6× His-tagged Cmr5α. The *cmr2α* gene fragment was inserted into pMAL-C5X-His between the Nde I and EcoR I restriction sites, such that the encoded proteins have a C-terminal 6× His tag and an N-terminal MBP tag. The *csx1* gene fragment was inserted into pET30a between the Nde I and Xho I restriction sites to generate C-terminal 6× His-tagged Csx1. The *csx1* gene fragment was inserted into pACYC_Duet-1 between the Nde I and Xho I restriction sites to generate C-terminal 6× His-tagged Csx1. All the primers were synthesized by Sangon Biotech (Shanghai, China). All the plasmids were confirmed by sequencing before transformation and then transformed to *E. coli* BL21 (DE3) to obtain corresponding strains. The strains are listed in Supplementary Table S1 and the plasmids are listed in Supplementary Table S2.

### Construction of *S. Islandicus* interference plasmids and strains

The pAC-SS1 was used to construct a series of interference plasmids. The expression cassette of *BHS13*_*gp06*, *BHS13_gp39* and *BHS13_gp40* were inserted in pAC-SS1, between Nde I and Nhe I, respectively (Supplementary Table S2). The plasmids targeting SMV1 were constructed in a similar way, except the spacer was replaced by sequences in SMV1 to interference infected SMV1. The expression cassette of AcrIIIB2 was inserted in pTSMV1_01, pTSMV1_04, pTSMV1_05, pTSMV1_36 and pTSMV1_38, respectively, generating pTSMV1_01OEAcrIIIB2, pTSMV1_04OEAcrIIIB2, pTSMV1_05OEAcrIIIB2, pTSMV1_36OEAcrIIIB2 and pTSMV1_38OEAcrIIIB2. The detailed procedures of *S. islandicus* E233S1 cultivation and transformation were described previously (29). All the primers were synthesized by Sangon Biotech (Shanghai, China). All the plasmids were confirmed by sequencing before transformation. The strains are listed in Supplementary Table S1 and the plasmids are listed in Supplementary Table S2.

### Protein expression and purification

To express His-tagged AcrIIIB2, Cmr4α, Cmr5α, MBP and MBP-Cmr2α-His from *E. coli* BL21 (DE3), the strains carrying the indicated plasmids were grown in LB medium containing the corresponding antibiotics. At an optical density optical density at 600 nm (OD_600_) of ∼1.0, protein expression was induced with 0.1 mM isopropyl-β-d-thiogalactopyranoside (IPTG) at 18°C for 18 h. Then, the cells were collected by centrifugation at 7000*g* for 10 min. The cell mass was resuspended in 50 ml of lysis buffer (50 mM Tris pH 8.0, 20 mM imidazole, 500 mM NaCl) and stored at −80°C until protein purification. To prepare cell extracts, the cells were disrupted by French press and the lysate was subjected to centrifugation at 13 000*g* for 40 min to remove cell debris. Then, the cell extract was loaded onto Ni-NTA agarose resin columns (Cytiva, Marlborough, MA, USA). After the column was washed with lysis buffer containing 60 mM imidazole, His-tagged proteins were eluted using a lysis buffer containing 300 mM imidazole. Then, the elution fractions were concentrated employing an Amiconultra centrifugal filter with corresponding cutoff (Millipore, Billerica, MA, USA) and then diluted for 30-fold with Buffer A (25 mM Tris-HCl, pH 8.0). The diluted samples were loaded onto a 5 ml HiTrap Q HP column (Cytiva, Marlborough, MA, USA) and the proteins were eluted using a 35 ml linear gradient of 0–1 M NaCl. The fractions containing target proteins were concentrated again and loaded onto a Superdex 200 Increase 10/300 GL column (Cytiva, Marlborough, MA, USA). Finally, the proteins were eluted with Buffer C (20 mM HEPES, pH 7.5, 250 mM NaCl) and analyzed by sodium dodecyl sulfate–polyacrylamide gel electrophoresis (SDS–PAGE). His-tagged Csx1 was purified in a similar way except the strains were grown in ZYP-5052 medium for auto-induction (30).

To express GST and GST-tagged AcrIIIB2, the strains carrying the indicated plasmids were grown in LB medium containing the corresponding antibiotics. The cells were harvested, lysed and the cell supernatant was collected as described before. Then, the cell extract was loaded onto Glutathione Sepharose 4B resin columns (Cytiva, Marlborough, MA, USA). After the column was washed with binding buffer, GST-tagged proteins were eluted using an elution buffer (50 mM Tris-HCl, 10 mM reduced glutathione, pH 8.0). Then, the elution fractions were concentrated employing an Amiconultra centrifugal filter with corresponding cutoff (Millipore, Billerica, MA, USA) and samples were loaded onto a 5 ml HiTrap Desalting column (Cytiva, Marlborough, MA, USA). Finally, the proteins were eluted with Buffer C (20 mM HEPES pH 7.5, 250 mM NaCl) and analyzed by SDS–PAGE.

To obtain nucleic acid-free AcrIIIB2, cell culture carrying pET28a-SUMO-AcrIIIB2 was grown in LB medium at 37°C until the OD_600_ reached 0.8–1.0. Expression was induced by adding IPTG to a final concentration of 0.5 mM at 16°C overnight. Cells were harvested by centrifugation (7000*g* for 10 min) and lysed by high-pressure homogeniser (800 bar) in buffer D (50 mM HEPES, pH 7.5, 10% (v/v) glycerol, 20 mM imidazole and 1 M NaCl). The lysate was centrifuged at 13 000*g* for 60 min at 4°C, and the supernatant was applied onto the Ni-NTA column (GE Healthcare) pre-equilibrated with buffer D. After washing with 20 column volumes of buffer D, the Ni-NTA column was washed at room temperature with 10 column volumes of buffer E (20 mM HEPES, pH 7.5, 4.5 M NaCl) to remove the nucleic acids retained by the AcrIIIB2, followed by 5 volumes of buffer D. The protein was eluted with buffer F (50 mM HEPES, pH 7.5, 1 M NaCl, 10% (v/v) glycerol and 300 mM imidazole). Then, the elution fractions were concentrated employing an Amiconultra centrifugal filter with corresponding cutoff (Millipore, Billerica, MA, USA) and loaded onto a Superdex 200 Increase 10/300 GL column (Cytiva, Marlborough, MA, USA) pre-equilibrated with buffer G (20 mM HEPES, pH 7.5, 150 mM NaCl and 10% (v/v) glycerol). Eluted proteins were incubated with SUMO-protease at 4°C overnight. After cleavage, the samples were incubated at 70°C for 20 min to remove SUMO protease. The His-6 -SUMO-tag cleaved AcrIIIB2 was further purified by Ni-NTA resin (QIAGEN) equilibrated with buffer G (20 Mm HEPES, pH 7.5, 150 mM NaCl and 10% (v/v) glycerol), the flow through fractions were pooled, concentrated by ultrafiltration and then snap-frozen in liquid nitrogen for later usage.

The MBP-tagged AcrIIIB2 was purified using high salt buffer (buffer D to buffer F) to remove nucleic acids and further purified using Superdex 200 Increase 10/300 GL column (Cytiva, Marlborough, MA, USA) pre-equilibrated with buffer G (20 mM HEPES, pH 7.5, 150 mM NaCl and 10% (v/v) glycerol).

The Cmr-α RNP was expressed as described before (31). *S. islandicus* strains carrying a *cmr6α* expression plasmid were grown in SCV at 78°C up to OD_600_ = 0.7, and cells were collected from at least 12 l of culture by centrifugation at 8000*g* for 10 min. The cell pellet was re-suspended in Buffer I (20 mM HEPES pH 7.5, 30 mM Imidazole, 500 mM NaCl) and disrupted by a French press. The cell extract was loaded onto a 1 ml HisTrap HP (GE Healthcare) and His-tagged protein was eluted by Buffer J (20 mM HEPES pH 7.5, 500 mM Imidazole, 500 mM NaCl). Five milliliters of Buffer B fractions were concentrated and further purified by size exclusion chromatography in Buffer K (20 mM Tris-HCl pH 7.5, 250 mM NaCl) with a Superdex 200 Increase 10/300 GL column (Cytiva, Marlborough, MA, USA). Sample fractions were analyzed by SDS–PAGE and those containing the complete set of Cmr–α components were pooled together and used for further analysis.

### β-galactosidase assay

β-glycosidase assay was carried out using ONPG (ρ-nitrophenyl-β-d-galactopyranoside) method as described previously (32). Transformants of reporter plasmids were grown in the indicated media and exponentially growing cells (OD_600_ = 0.2–0.3) were collected and re-suspended in 10 Mm Tris-HCl, pH 8.0. The cell suspensions were sonicated to yield crude cellular extracts. After removing cell debris by centrifugation at 13 000*g* and 4°C for 30 min), the cell lysates were suitable for ONPG assay. Protein contents of the cellular extracts were determined using Micro BCA Protein Assay Kit (Pierce). Samples were either assayed immediately or stored at −80°C. For each assay, 10–50 μl of supernatant was transferred to a microcentrifuge tube containing 450–490 μl of reaction buffer with 2.8 mM ONPG and 50 mM sodium phosphate, pH 6.5, in a total volume of 500 μl. Samples were incubated at 75°C for 10 min and the reaction was stopped by adding an equal volume of 1 M sodium carbonate. Yields of 4-nitrophenol were determined by measuring at 420 nm using Nanodrop1000 Spectrophotometers. One specific unit was defined as 1 mmol 4-nitrophenol produced per min per mg total protein.

### Total RNA extraction and reverse transcriptase with quantitative polymerase chain reaction

Total RNA samples to be used for reverse transcriptase with quantitative polymerase chain reaction (qPCR) analysis were extracted using TRIzol® (Invitrogen) according to the manufacturer's instructions. Then, 1 μg RNA was used for generating complementary DNA (cDNA) with the HiScript® II Q RT SuperMix for qPCR (+gDNA wiper) (Vazyme, Nanjing, China) using random primer in a 10-μl volume. Two primer sets, Qtarget and Qref that were designed for amplification of PCR products in the target region or in a reference region of the mRNA, respectively, were employed for PCR amplification using Taq Pro Universal SYBR qPCR Master Mix (Vazyme, Nanjing, China) and a QuantStudio 5 Real-Time PCR System (Applied Biosystem) with the following PCR condition: denaturing at 95°C for 5 min, 40 cycles of 95°C for 15 s, 60°C for 20 s and 72°C for 20 s. The amount of uncut mRNA is expressed as the ratio between the amount of S1 RNA protospacer in pAC-SS1-Virus gene transformants and that in the corresponding pAC-SS1 and pSeSD1 transformants.

### Virus propagation

A sensitive strain, *S. islandicus* ΔC1C2 (33), was grown at exponential phase for at least 72 h. At an OD_600_ of ∼0.2, 100 ml of the culture was infected with SMV1 at a MOI of <0.1. At 48 h postinfection (hpi), the cells were removed by centrifugation at 8000*g* for 10 min and the virus particles in the supernatant were concentrated by ultrafiltration using 1 000 000 molecular-weight-cutoff Amicon ultra centrifugal filter (Millipore, Billerica, MA, USA). The concentrated virus particles were then dissolved in viral storage buffer containing 20 mM Tris-HCl, pH 7.0, 20% glycerol and stored at 4°C before use.

### Plaque forming unit assay

SCVU plates were prepared before the assay. To determine viral titer in the storage buffer, the storage buffer was serially diluted and mixed with 4 ml of fresh *S. islandicus* ΔC1C2 culture (OD_600_ around 0.4). The mixture was preheated at 75°C and further mixed with an equal volume of preheated 0.4% GELRITE (Duchefa-biochemie, Haarlem, Netherlands). Then, the mixture was spread onto pre-warmed SCVU plates. Plaques were counted after the plates had been incubated at 75°C for 2 days.

To perform the drop plaque assay, ΔC1C2 plates were firstly prepared by spreading the mixture of 4 ml of preheated *S. islandicus* ΔC1C2 culture (OD_600_ around 0.4) and 4 ml of preheated 0.4% GELRITE and 100 μl of Mg^2+^/Ca^2+^ onto SCVU plates. Then, cells were removed from the SMV1-infected cultures and the supernatant was serially diluted. Then, 10 μl of the diluted supernatant (from 10^−1^ to 10^−6^) was dropped on the ΔC1C2 plates. Pictures of the plates were taken at corresponding time.

### Viral infection and sampling

The strains were grown at exponential phase for at least 72 h. At an OD_600_ of ∼0.1, 50 ml of the cultures were infected with SMV1 at indicated MOI. The MOI was calculated based on the estimation that 1 ml of OD_600_ = 0.1 culture contains 1 × 10^8^ cells. At indicated time points, samples were removed from the cultures for analysis.

### RNA and ssDNA cleavage assays

RNA and ssDNA cleavage assays performed with Cmr-α RNP were conducted as described previously (31). RNA cleavage assay performed with Csx1 was conducted as described previously. RNA and ssDNA substrates were labeled with 5′-end FAM and synthesized at Takara (Dalian, China). For target RNA cleavage assays in Figure 5, the reaction mixture (10 μl in total) contains 50 nM sole Cmr-α or AcrIIIB2-bound Cmr-α complex and the indicated concentration of target RNA substrates in the buffer (20 mM MES, 1 mM MnCl_2_, 1 mM dithiothreitol, pH 6.0). The reaction was performed at 70°C and stopped at the indicated time point by the addition of 2× RNA loading dye (NEB) and cooling on ice. Finally, the samples were heated for 5 min at 95°C and separated on denaturing polyacrylamide gels (18% polyacrylamide) and visualized by fluorescence imaging using a Typhoon FLA 7000 laser scanner (GE Healthcare). For ssDNA cleavage assay in Figure 5, the mixture (10 μl in total) contains 50 nM sole Cmr-α or AcrIIIB2-bound Cmr-α complex, 50 nM or 1 μM target RNA, 250 nM ssDNA substrate in the buffer (20 mM MES, 1 mM MnCl_2_, 1 mM dithiothreitol, pH 6.0). The reaction was performed at 70°C and the reaction was taken out and kept on ice at the indicated time point, reactions were stopped by adding 1 μl of 6 M guanidinium thiocyanate and 10 μl of 2× RNA loading dye (NEB). The samples were heated for 5 min at 95°C and separated on 18% denaturing polyacrylamide gels and visualized by fluorescence imaging. For AcrIIIB2-His protein related cleavage assays, the reaction performed the same except the amount of AcrIIIB2-His added.

Quantification analysis of the RNA and ssDNA cleavage assays were performed with ImageJ as described previously (34).

### Pull-down assay

For the *in vitro* pull-down assay, His-tagged Cmr-α complex, Cmr4α, Cmr5α, Csx1, MBP or MBP-Cmr2α-His was incubated with GST-tagged AcrIIIB2 and GST, respectively, in 500 μl of assay buffer (140 mM NaCl, 2.7 mM KCl, 10 mM Na_2_HPO_4_, 1.8 mM KH_2_PO_4_, pH 7.3 0.1% Triton X-100) at 4°C for 6 h. The proteins were then mixed with 50 μl of Ni SepharoseTM 6 Fast Flow (Cytiva) with constant rotation at 4°C for 1 h. The beads were then washed thrice with assay buffer containing 20 mM imidazole. Subsequently, beads were boiled in loading buffer and the pulled-down protein complexes were separated on 15% SDS–PAGE.

For the *in vivo* pull-down assay, for co-purification of His-tagged Cmr4α, Cmr5α or Csx1 and non-tagged AcrIIIB2, plasmids expressing Csx1 and AcrIIIB2 were transformed into *E. coli* BL21(DE3) cells, which were cultured in 500 ml of LB medium containing 25 μg/ml chloramphenicol at 37°C for 3 h to an OD_600_ of 0.6–0.7, and then induced with 0.1 mM IPTG for 16 h at 12°C. The cells were harvested and resuspended, then lysed by ultrasonication. Supernatant was collected after centrifugation and filtrated with 0.22-μm filter. The filtered supernatant was loaded onto a Ni-NTA agarose column. The column was washed with 10 volumes of elution buffer (50 mM Tris-HCl pH 8.0, 300 mM NaCl and 20 mM Imidazole) and protein eluted by gradient imidazole solution. Samples collected at each step were analysis by SDS–PAGE.

### Western blot

Proteins from SDS–PAGE gels were transferred onto a PVDF membrane (Bio-Rad, Hercules, CA, USA) using Trans-Blot SD Semi-Dry Transfer Cell (Bio-Rad, Hercules, CA, USA). The membrane was blocked in TBST buffer (50 mM Tris, 100 mM Nacl, 0.05% Tween 40, pH 8.0) containing 6% milk, followed by incubation with anti-GST serum. On completion of three consecutive washing steps, the membrane was incubated with the secondary antibody (Goat Anti-Mouse IgG) (Proteintech, Wuhan, China). After removing unspecific binding, the Clarity Western ECL Substrate (Bio-Rad, Hercules, CA, USA) was dropped onto the membrane and the signals were recorded using Tanon 5200 (Tanon, Shanghai, China).

### Electrophoretic mobility shift assay

To analyze the nucleic acid affinity of AcrIIIB2 and effects of AcrIIIB2, four different FAM labeled RNA substrates (RNA1, RNA2, RNA3 and RNA4) and one FAM labeled ssDNA substrate (ssDNA1) were incubated with His-tagged AcrIIIB2, respectively, in a 10-μl mixture at 70°C for 5 min. The incubation buffer contained 20 mM Mes pH 6.0, 10 mM EDTA. The sequences of the substrates are listed in Supplementary Table S4. The concentration of the substrates was fixed as 250 nM, while the concentrations of proteins varied as indicated in the figure legends. After incubation, the reaction samples were mixed with 2× RNA loading dye (NEB) and loaded onto 12% native polyacrylamide gels. The electrophoresis was performed in 0.5× TB buffer (44.5 mM Tris, 44.5 mM boric acid) at 200 V for 40 min. Last, the fluorescent signal was visualized using a Fujifilm FLA-5100 scanner (Fujifilm Life Science, Japan).

To analyze the effect of AcrIIIB2 on the interaction between Cmr-α RNP and the target-RNA, 25 nM Cmr-α RNP, indicated concentrations of AcrIIIB2 were incubated with 250 nM RNA1 in the electrophoretic mobility shift assay (EMSA) buffer at 70°C for 10 min. Last, the samples were analyzed by native PAGE and visualized with a Fujifilm scanner.

To detect the effect of AcrIIIB2 on the binding between cOAs and Csx1 protein, we synthesized the α-cOAs using the α-^32^P labeled ATP substrates. Then, the α-cOAs and Csx1 were incubated at 70°C for 5 min with or without AcrIIIB2 addition, and the reactions were stopped by addition of 2× RNA loading dye. After cooling on ice for 5 min, all samples were separated on 10% native PAGE. Labeled products were detected by exposing the gel to a phosphor screen and scanned with a Fujifilm scanner (FLA-5100).

### Analysis of cOA synthesis

Biochemical assays for testing cOA synthesis from α-^32^P labeled ATP by Cmr-α RNP were conducted as previously described (34). To detect the effect of AcrIIIB2 on cOA synthesis, Cmr-α RNP with the target RNA was incubated with AcrIIIB2 before addition of α-^32^P labeled ATP substrate. Then, the samples were incubated at 70°C for different incubation times, and the reactions were stopped by addition of 10 μl of 2× RNA loading dye. After denaturing by heating to 95°C for 2 min and cooling on ice for 5 min, all samples were separated on 24% denaturing PAGE. Labeled products were detected by exposing the gel to a phosphor screen and scanned with a FUJIFILM scanner (FLA-5100).

### Comparison of viral genomes

Visualization of genomic comparison was done with EasyFig (35).

## Results

### SIRV3 encodes a novel acr to inhibit type III-B immunity *in vivo*

**Figure 1.**
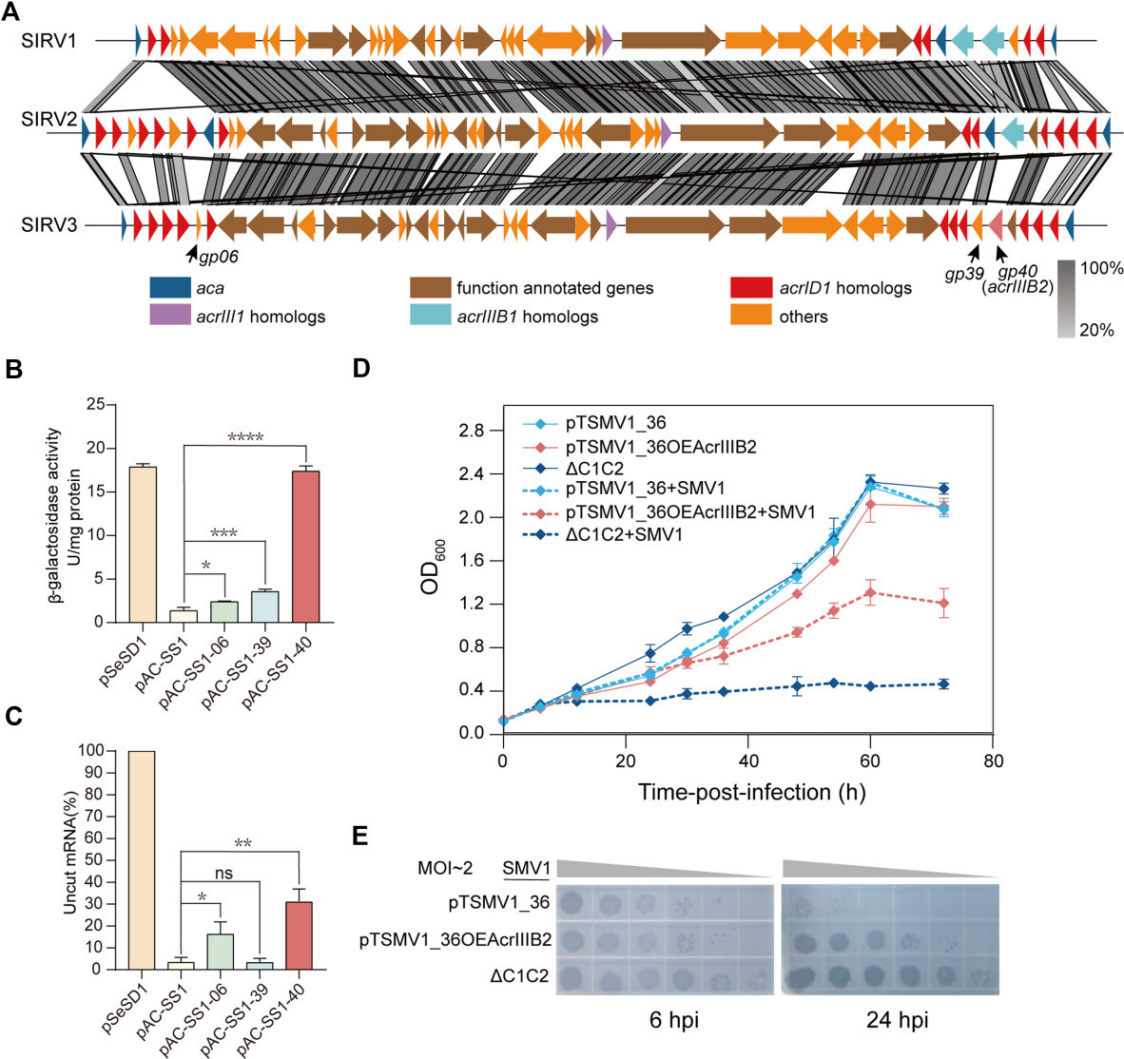
AcrIIIB2 inhibits the immunity of Type III-B CRISPR–Cas systems in *S. islandicus*. (**A**) Genome comparison between *Sulfolobus* viruses SIRV1, SIRV2, and SIRV3. The *acrID1*, *acrIIIB1*, *acrIII1* and their corresponding homologs are colored red, light blue and purple, respectively. The anti-CRISPR-associated protein genes (*aca*) are colored blue. The genes encoding function-annotated proteins are colored brown and other genes are colored orange. The black arrows point to *BHS13_gp06*, *BSH13_gp39* and *BHS13_gp40* genes. (**B**) Specific β-glycosidase activities of E233 transformants carrying empty vector (pSeSD1), a targeted plasmid (pAC-SS1) or pAC-SS1 overexpressing *gp06*, *gp39* or *gp40* genes, respectively. (**C**) Uncut *lacS* mRNA levels of E233 transformants carrying different plasmids. Three independent transformants of each construct were analysed for β-glycosidase assay as well as mRNA quantification (unpaired *t*-test; *P < 0.05; **P < 0.01; ***P < 0.001; ****P < 0.0001; Bars represent mean ± SD). (**D**) Growth curves of E233 strains containing plasmids and ΔC1C2 strain with or without SMV1 infection. pTSMV1_36: plasmid containing spacer targeting the early gene *CF87_gp36* gene of SMV1; pTSMV1_36OEgp40: plasmid pTSMV1_36 cloned with the SIRV3 *BHS13_gp40* gene expression cassette. The data show means of three replicates. Error bars indicate the standard deviations. (**E**) Plaques of the supernatant of SMV1-infected-cultures at 6 hpi and 24 hpi from (**D**) on the plates spread with ΔC1C2 cells.

In this study, we compared the genome sequence of the *Sulfolobus* virus SIRV3, SIRV1 (27) and SIRV2 (28) using Easyfig (35) to detect unidentified anti-CRISPR (*acr*) genes (Figure 1A). Based on previously reported Acr characteristics (26), we focused on three small genes with unknown functions (*BHS13_ gp06*, *BHS13_ gp39*, and *BHS13_ gp40*), which were located near the two putative Acr-associated-genes (*aca*) *BHS13_ gp01* and *BHS13_ gp45* (36) (Figure 1A). First, we examined the effect of the Acr candidates on the RNA interference activity of Type III-B system through analysing the targeted reporter gene transcript and activity. The E233 strain carrying blank control plasmid showed high β-galactosidase activity and high *lacS* transcript level, while that carrying interference plasmid pAC-SS1 targeting the *lacS* transcript exhibited opposite results (Figure 1B and C). Expression of *gp06* and *gp40* gene significantly increased both β-galactosidase activity (*P* < 0.05 and *P* < 0.0001, respectively) and mRNA level (*P* < 0.05 and *P* < 0.01, respectively), indicating the inhibition effect of the *gp06*- and *gp40*-encoded proteins on Type III-B RNA interference activity (Figure 1B and C). Expression of *gp39* significantly increased (*P* < 0.001) the β-galactosidase activity (Figure 1B) but merely increased mRNA level (Figure 1C), implying slight inhibition of Type III-B immunity. *S. islandicus* E233 encoded two modules of Type III-B CRISPR–Cas systems, namely, Cmr-α and Cmr-β. Next, we investigated the effects of the three Acr candidates on RNA cleavage activities of Cmr-α strain (E233 ΔI-AΔCmr-β strain, encoded Cmr-α module only). We obtained similar results that expression of *gp40* gene showed significantly inhibitory effect on Type III-B Cmr-α module but expression of *gp06* gene did not (Supplementary Figure S1B and C).

Next, we investigated the inhibitory effect of *gp40*-encoded protein on Type III-B immunity against viruses. Since SIRV3 could cause a host-dependent carrier state infection in *S. islandicus* REY15A (37), we used a *Sulfolobus* virus SMV1 that can induce culture growth retardation and form clear visible plaques in *S. islandicus* REY15A (38). Cultures containing a SMV1-targeting plasmid pTSMV1_36 and cultures containing the plasmid pTSMV1_36 cloned with *gp40* overexpression cassette (pTSMV1_36OE40) were infected by SMV1 at a multiplicity of infection (MOI) of ∼2, using ΔC1C2 strain that lacks any CRISPR–Cas immunity as the control strain. Cultures containing pTSMV1_36 showed the same growth state with or without viral infection, while cultures containing pTSMV1_36OE40 and control strain ΔC1C2 displayed significant growth retardation after viral infection (Figure 1D). Moreover, plaque forming units of the supernatant of cultures containing pTSMV1_36OE40 were significantly higher than the supernatant of cultures containing pTSMV1_36 (Figure 1E and Supplementary Figure S1D). Together, these results indicate that *gp40* encodes an anti-CRISPR protein showing strong inhibitory effect on Type III-B immunity in *S. islandicus*, and we name it AcrIIIB2 in this study.

### AcrIIIB2 enables SMV1 to escape Cmr-α immunity targeting both early and middle/late genes

**Figure 2.**
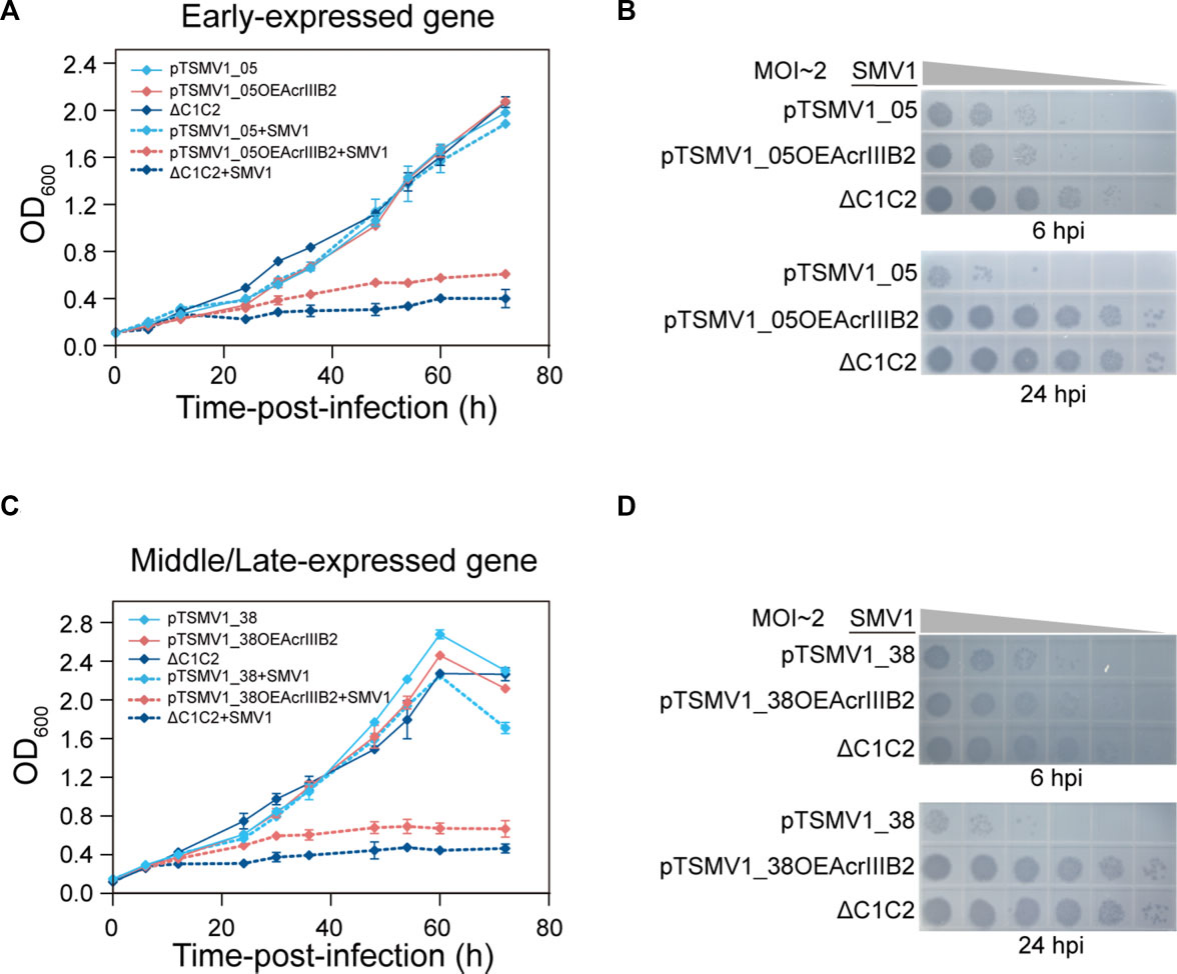
AcrIIIB2 inhibits Cmr-α immunity targeting early-expressed or middle/late-expressed viral genes. Targeting of SMV1 early-expressed gene (**A**) or middle/late-expressed gene (**C**) by Cmr-α strain carrying the plasmid pTSMV1_05 or pTSMV1_38 is inhibited by AcrIIIB2 at MOI ∼2. (**B**) Plaques of the supernatant of SMV1-infected-cultures sampled at 6 and 24 hpi from (**A**) or (**D**) 6 and 24 hpi from (**C**) on the plates spread with ΔC1C2 cells. The data show means of three replicates. Error bars indicate the standard deviations.

Here, we investigated the inhibitory effect of AcrIIIB2 on the immunity of Type III-B Cmr-α modules programmed to target early-expressed genes (*CF87_gp01* and *CF87_gp05*) and middle-/late-expressed genes (*CF87_ gp04* and *CF87_ gp38*) of SMV1. Both interference plasmids and that carrying *acrIIIB2* gene expression cassette were transformed into Cmr-α strain and the transformant cultures were infected by SMV1 at a MOI of ∼2. Without SMV1 infection, the cultures in presence or absence of *acrIIIB2* gene showed the same growth curves compared with the ΔC1C2 control cultures in SCV medium (Figure 2A and C). After being infected, the cultures containing interference plasmids showed strong immunity against SMV1 while the cultures expressing AcrIIIB2 showed severe growth retardation (Figure 2A and C; Supplementary Figure S2A and C), regardless of the viral early or middle/late genes to be targeted. Moreover, the virus titer in the supernatant of the transformant cultures that expresses AcrIIIB2 was 10- to 10^5^-fold more than that in the cultures without AcrIIIB2 expression (Figure 2B and D; Supplementary Figure S2B and D). These results showed the strong inhibitory effect of AcrIIIB2 on Type III-B Cmr-α immunity regardless of targeting viral early or middle/late genes.

To further investigate the inhibitory capability of AcrIIIB2, we infected these strains at a low MOI of ∼0.01 or an extremely low MOI of ∼10^−6^. We observed the same results that AcrIIIB2 inhibits Cmr-α immunity targeting early-expressed (Supplementary Figures S3A–C and S4A–C) or middle-/late-expressed viral genes (Supplementary Figures S3D–F and S4D–F). The cultures of cells containing interference plasmid expressing AcrIIIB2 and infected with SMV1 showed significant growth retardation and viral titers in the supernatant was 10- to 10^5^-folds more than that in the cultures of cells only carrying the interference plasmid at MOI of ∼0.01 (Supplementary Figure S3). We did not observe growth retardation of the corresponding cultures at MOI of ∼10^−6^ but viral titers of the supernatant of the cultures which carry interference plasmid expressing AcrIIIB2 was 10^3^–10^5^-fold more than that in the cultures only carrying the interference plasmid (Supplementary Figure S4). Taken together, our results demonstrated that AcrIIIB2 inactivated Cmr-α immunity regardless of targeting early or middle/late viral genes.

### AcrIIIB2 binds cmr4α subunit and inhibits *cis* RNase and *trans* ssDNase activities of Cmr-α

**Figure 3.**
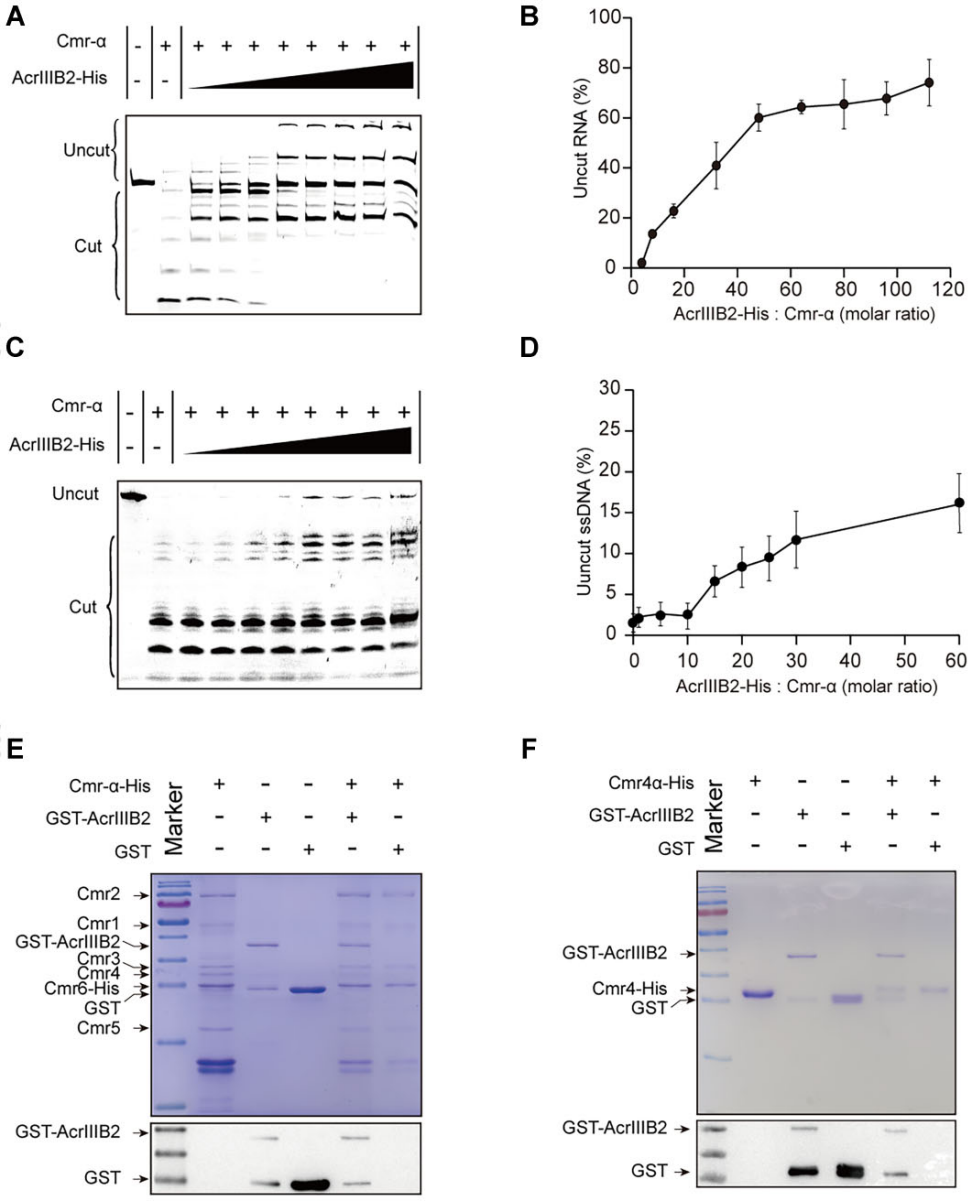
AcrIIIB2 interacts with Cmr4α to inhibit the RNase and ssDNase activities of Cmr-α RNP. (**A**) AcrIIIB2 inhibits *cis* RNase activity of Cmr-α RNP. Cmr-α RNP (100 nM), AcrIIIB2 (at different concentrations), 250 nM labeled target RNA were co-incubated for 5 minutes at 70°C at AcrIIB2 to Cmr-α RNP molar ratio of 0, 1, 5, 10, 15, 20, 25 and 30. (**B**) Dose response of Cmr-α RNP *cis*-RNase inhibition by AcrIIIB2. (**C**) AcrIIIB2 inhibits *trans* ssDNase activity. Cmr-α RNP (100nM), AcrIIIB2 (at different concentrations), unlabeled target RNA (300 nM) and 5′-FAM-labeled ssDNA (300 nM) were co-incubated for 5min at 70°C at AcrIIIB2 to Cmr-α RNP molar ratios of 0, 1, 5, 10, 15, 20, 25 and 30. (**D**) Dose response of Cmr-α RNP *trans*-ssDNase inhibition by AcrIIIB2. (**E**) His-tag pull-down assays of GST-AcrIIIB2 with Cmr-α RNP complex. GST-AcrIIIB2, GST and Cmr-α subunit proteins are indicated by black arrows (top). Western blot assays were performed to show GST-tagged AcrIIIB2 using the anti-GST antibody. (**F**) His-tag pull-down assays of GST-AcrIIIB2 with Cmr4α subunit. GST-AcrIIIB2, GST and Cmr4α-His proteins are indicated by black arrows (top). Western blot assays were performed to show GST-tagged AcrIIIB2 using the anti-GST antibody.

*S. islandicus* Type III-B CRISPR system possesses multiple functions, including *cis* RNA cleavage, *trans* ssDNA cleavage, cOA synthesis and collateral RNA cleavage by the associated Csx1 (39). To examine the inhibitory effects of AcrIIIB2 on Type III-B CRISPR–Cas system, we purified all relevant proteins. The Cmr-α RNP purified from *S. islandicus* exhibited strong *cis* RNA cleavage activity against a 43 nt 5′-end FAM-labeled RNA substrate matching the crRNA, and this activity was gradually decreased with addition of increasing amount of AcrIIIB2 (Figure 3A). Quantification results of the signal density of the uncleaved RNA target indicated that ∼80% target RNA was protected at the AcrIIIB2-to- Cmr-α RNP ratio of 30 (Figure 3B). Further, we tested the inhibitory effect of AcrIIIB2 on *trans* ssDNase activity of Cmr-α RNP. In presence of the target RNA, Cmr-α RNP cleaved 5′-end FAM-labeled 59 nt ssDNA into short fragments (Figure 3C), and the addition of AcrIIIB2 slightly inhibited ssDNA cleavage (Figure 3C). Quantification results of the signal density of the uncleaved ssDNA substrate showed that ca.15% ssDNA was protected by AcrIIIB2 (Figure 3D).

To reveal the inhibitory mechanism of AcrIIIB2 in detail, we first investigated the interactions between AcrIIIB2 and Cmr-α complex by pull-down assay. GST-tagged AcrIIIB2 did not bind Ni-NTA beads (data not shown). Our pull-down data showed that GST-AcrIIIB2 was co-purified with His-tagged Cmr-α RNP by Ni-NTA beads, while GST as the control was not co-purified (Figure 3E). Western blotting assay using anti-GST antibody also confirmed that GST-AcrIIIB2 bound His-tagged Cmr-α, but GST did not (Figure 3E). Cmr-α complex consists of six subunits, of which Cmr4α and Cmr5α subunits containing multiple copies to form the main backbone (40,41). Moreover, the conserved aspartic acid of Cmr4α is the vital site for ruler cleavage of target RNA (40,42–44). Based on this, we next investigated the interaction between AcrIIIB2 with Cmr4α and Cmr5α individually. The pull-down assay results showed that GST-AcrIIIB2 was indeed co-purified with His-tagged Cmr4α, while GST was not (Figure 3F), which was further supported by western blotting assay using anti-GST antibody (Figure 3F). We also confirmed the interaction between Cmr4α with AcrIIIB2 both *in vitro and in vivo* using nucleic acids-free AcrIIIB2. We noticed that AcrIIIB2 carried a large amount of nucleic acids (Supplementary Figure S8A). To exclude the influence of nucleic acids, we purified MBP-tagged AcrIIIB2 using high salt buffer to remove nucleic acids. Next, we incubated MBP-tagged AcrIIIB2 and Cmr4α-His at 37°C and then performed the gel filtration assay. The results showed that Cmr4α-His were co-eluted with MBP-AcrIIIB2 (Supplementary Figure S5A and D) in fractions that solo Cmr4α-His was not eluted (Supplementary Figure S5G). MBP-tag was used as a control to exclude the binding between MBP-tag with Cmr4α-His (Supplementary Figure S5C and E). To confirm the interaction *in vivo*, we cloned AcrIIIB2 and Cmr4α into pACYC_Duet-1 in which Cmr4α fused with a C-terminal 6× His tag and AcrIIIB2 did not fused with any tags. AcrIIIB2 was co-eluted with Cmr4α-His in the fraction contained 300 mM imidazole (Supplementary Figure S6), which indicated that AcrIIIB2 interacts with Cmr4α *in vivo*. Our pull-down assay further showed that another backbone subunit Cmr5α was not co-purified with GST-AcrIIIB2, suggesting that Cmr5α could not bind AcrIIIB2 directly (Supplementary Figure S6A). The HD domain of Cas10 subunit is responsible for non-specific ssDNA cleavage. However, we did not detect the direct interaction between AcrIIIB2 and Cas10 subunit in our pull-down assay (Supplementary Figure S6B). The *in vivo* pull-down experiment also supported that Cmr5α-His did not interact with AcrIIIB2 (Supplementary Figure S7C and D).

### AcrIIIB2 binds RNA substrates non-specifically and forms super-complex with target-RNA and Cmr-α complex

**Figure 4.**
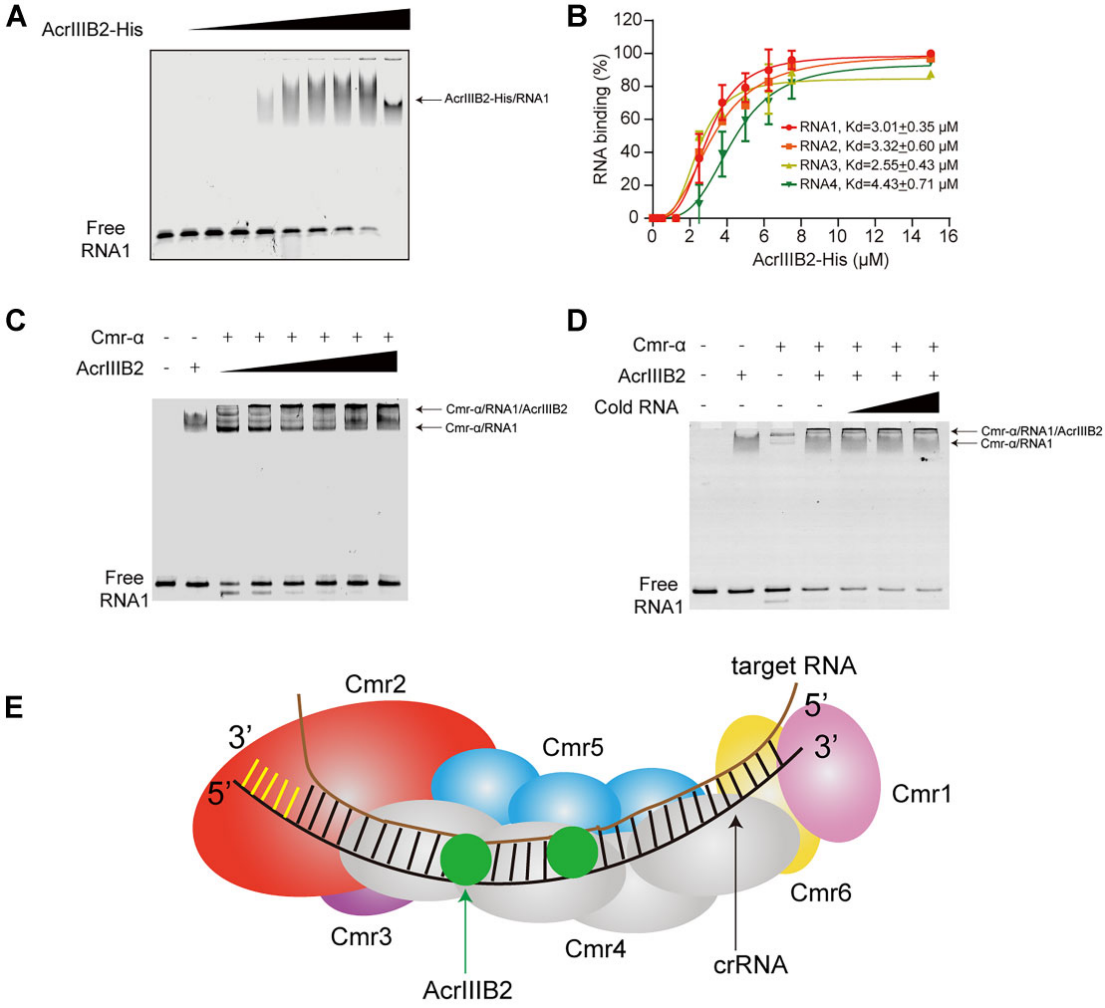
AcrIIIB2 bound with Cmr-α and target RNA to form a super-complex. (**A**) EMSA analysis of 5′-FAM labeled RNA substrate 1 with increasing amount of AcrIIIB2. 250 nM labeled target RNA were co-incubated for 5 min at 70°C with AcrIIIB2 (0, 0.25, 0.5, 1.25, 2.5, 3.75, 5, 6.25, 7.5 and 15 μM). (**B**) Quantified fractions of RNA substrates bound by AcrIIIB2 as determined by EMSA (mean ± SD, *n* = 3 independent experiments). (**C**) EMSA analysis of 5′-FAM labeled RNA substrate 1 with Cmr-α RNP in the presence of increasing amount of AcrIIIB2. Cmr-α RNP (250 nM), AcrIIIB2 (0, 0.25, 0.5, 1.25, 2.5 and 3.75 μM), 250 nM labeled target RNA were co-incubated for 5 min at 70°C at AcrIIIB2 to Cmr-α RNP molar ratio of 0, 1, 2, 5, 10 and 15. (**D**) EMSA analysis of 5′-FAM labeled RNA substrate 1 with Cmr-α RNP in the presence of increasing amount of cold non-target RNA substrates (RNA5). Cmr-α RNP (250 nM), AcrIIIB2 (250 nM), labeled target RNA (100 nM) and cold non-target RNA (100, 500 and 1000 nM) were co-incubated for 5 min at 70°C. (**E**) Model for AcrIIIB2- target RNA-Cmr-α super complex. AcrIIIB2 interact with intermediate Cmr4α and probably with target RNA.

We noticed that probable larger shifts formed with increasing amount of AcrIIIB2 in the RNA cleavage assays (Figure 3A), suggesting AcrIIIB2 could bind the RNA substrate firmly. So, we performed EMSA assays and confirmed that AcrIIIB2 bound with target RNA (Figure 4A), even at a much lower temperature (Supplementary Figure S8D). We also observed that no shifts formed in the ssDNA cleavage assay (Figure 3C) and confirmed AcrIIIB2 did not bind ssDNA in an EMSA assay (Supplementary Figure S8B). Moreover, we observed a high nucleic acid absorption peak (Supplementary Figure S8A) when we purified His-tagged AcrIIIB2 from *E. coli*, which suggests the RNA binding activity of AcrIIIB2 is non-specific. Then, we confirmed that AcrIIIB2 bound different RNA substrates (Supplementary Figure S8E) and showed no difference of affinity with these RNA substrates (Figure 4B). However, in RNase A cleavage experiment, we found that AcrIIIB2 failed to protect the RNA substrate (Supplementary Figure S8C). Further, we investigated the effect of AcrIIIB2 on the binding between Cmr-α RNP and the target RNA. Cmr-α RNP and AcrIIIB2 both bound with RNA substrates and formed different binding shifts, respectively (Figure 4C). With increasing amount of AcrIIIB2, a larger shift formed in the loading well (Figure 4C). This result suggests the larger shift is a large complex containing AcrIIIB2, Cmr-α and the target RNA. To explore how this complex is formed, we added cold non-target RNA into the reaction to compete with target RNA. However, a large amount of cold RNA did not displace the binding target RNA substrates (Figure 4D), suggesting the target RNA was bound by Cmr-α RNP through base-pairing with the crRNA and AcrIIIB2 was bound by Cmr-α RNP through binding with Cmr4α to form the super complex (Figure 4E).

### AcrIIIB2 inhibits target RNA cleavage probably through preventing dissociation of cleaved target RNAs from the Cmr-α complex

**Figure 5.**
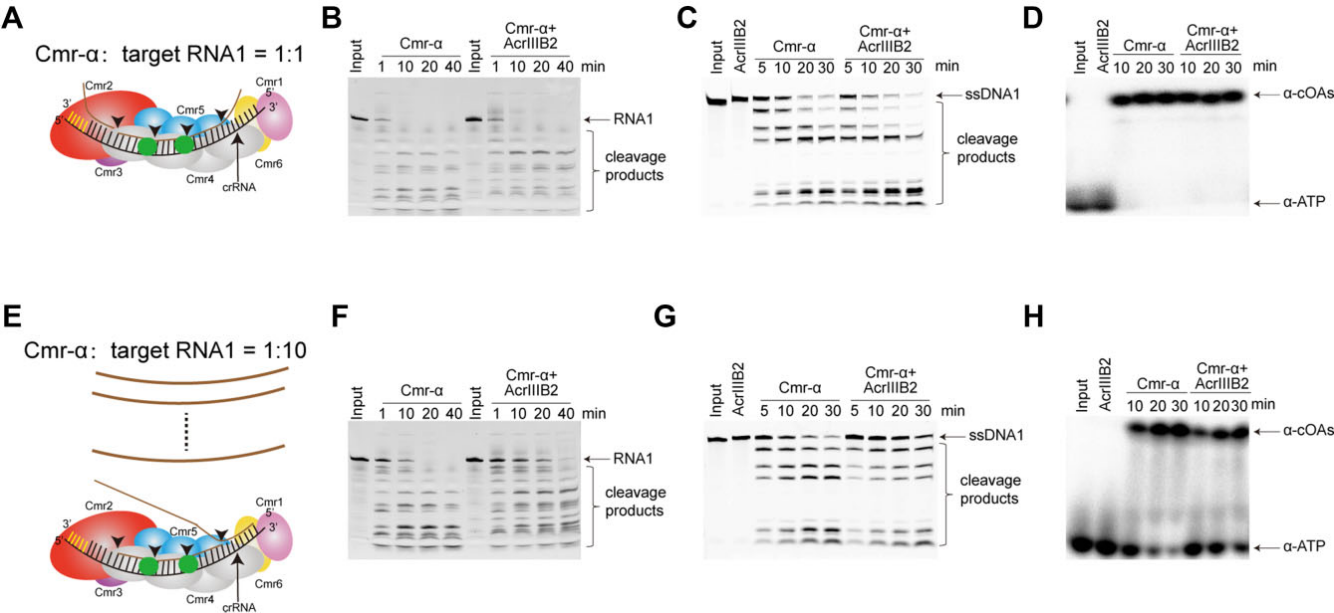
AcrIIIB2 inhibits Cmr-α turnover to reduce Cmr-α activities. (**A**) Diagram of target RNA cleavage when molar ratio of Cmr-α to target RNA equal to 1. (**B**) Target RNA cleavage by Cmr-α, and AcrIIIB2-bound Cmr-α complex at molar ratio of proteins to target RNA equal to 1. Cmr-α RNP (100nM) or Cmr-α-AcrIIIB2 (100 nM) were incubated with 100 nM 5′ FAM-labeled target RNA at 70°C for 1, 10, 20 and 40 min. Samples were separated by a 18% denaturing PAGE gel. (**C**) ssDNA cleavage by Cmr-α, or AcrIIIB2-bound Cmr-α complex at molar ratio of proteins to target RNA equal to 1. Cmr-α RNP (100 nM) or Cmr-α-AcrIIIB2 (100 nM) were incubated with 100 nM cold target RNA and 100 nM 5′ FAM-labeled ssDNA substrates at 70°C for 5, 10, 20 and 30 min. Samples were separated by a 18% denaturing PAGE gel. (**D**) COAs synthesis by Cmr-α, or AcrIIIB2-bound Cmr-α complex at molar ratio of proteins to target RNA equal to 1. Cmr-α RNP (100 nM) or Cmr-α-AcrIIIB2 (100 nM) were incubated with 100 nM cold target RNA and 100 μM cold ATP and radiolabeled α-ATP at 70°C for 10, 20 and 30 min. Samples were separated by a 24% denaturing PAGE gel. (**E**) Diagram of target RNA cleavage when molar ratio of Cmr-α to target RNA equal to 10. (**F**) Target RNA cleavage by Cmr-α, and AcrIIIB2-bound Cmr-α complex at molar ratio of proteins to target RNA equal to 10. Cmr-α RNP (25 nM) or Cmr-α-AcrIIIB2 (25 nM) were incubated with 250 nM 5′ FAM-labeled target RNA at 70°C for 1, 10, 20 and 40 min. Samples were separated by a 18% denaturing PAGE gel. (**G**) DNA cleavage by Cmr-α, or AcrIIIB2-bound Cmr-α complex at molar ratio of proteins to target RNA equal to 10. Cmr-α RNP (25 nM) or Cmr-α-AcrIIIB2 (25 nM) were incubated with 250 nM cold target RNA and 500 nM 5′ FAM-labeled ssDNA substrates at 70°C for 5, 10, 20 and 30 min. Samples were separated by a 18% denaturing PAGE gel. (**H**) COAs synthesis by Cmr-α, or AcrIIIB2-bound Cmr-α complex at molar ratio of proteins to target RNA equal to 10. Cmr-α RNP (25 nM) or Cmr-α-AcrIIIB2 (25 nM) were incubated with 250 nM cold target RNA and 1000 μM cold ATP and radiolabeled α-ATP at 70°C for 10, 20 and 30 min. Samples were separated by a 24% denaturing PAGE gel.

To further explore the mechanism that AcrIIIB2 inhibit Type III-B immunity, we measured the activities of Cmr-α and Cmr-α-AcrIIIB2 complex under different Cmr-α-to-target RNA molar ratios (Figure 5). We incubated the nucleic acid-free AcrIIIB2 with Cmr-α complex at 70°C for 20 min and then performed the gel filtration assay to get Cmr-α-AcrIIIB2 complex (Supplementary Figure S9B and C). With the relevant protein, we found that when molar ratio of the Cmr-α RNP to target RNA equal to one (Figure 5A), AcrIIIB2 had no effect on Cmr-α activities including RNA cleavage (Figure 5B), ssDNA cleavage (Figure 5C) and cOAs synthesis (Figure 5D). However, when molar ratio of the Cmr-α RNP to target RNA equal to 10 (Figure 5E), AcrIIIB2 reduced these Cmr-α activities (Figure 5F–H). Because AcrIIIB2 does not inhibit RNase activity of Cmr-α RNP (Figure 5B), these results suggest only two possibilities for inhibition of target RNA cleavage: (i) AcrIIIB2 inhibits the recognition of Cmr-α RNP to target RNA, and (ii) once the target RNA is cleaved by Cmr-α RNP, AcrIIIB2 inhibits the disassociation of cleaved target RNA, therefore, blocking the access of other target RNA substrates into Cmr-α RNP. Because we have confirmed that the recognition and binding between target RNA and Cmr-α RNP are based on the base-pairing between target RNA and crRNA in Cmr-α RNP (Figure 4D), leaving the last hypothesis as only possibility. In summary, at higher target RNA-to-Cmr-α molar ratio, the RNase, ssDNase and cOAs synthesis activities of Cmr-α were inhibited by AcrIIIB2 most probably through inhibiting the disassociation of cleaved target RNA from Cmr-α RNP.

### AcrIIIB2 has no direct effect on Csx1

**Figure 6.**
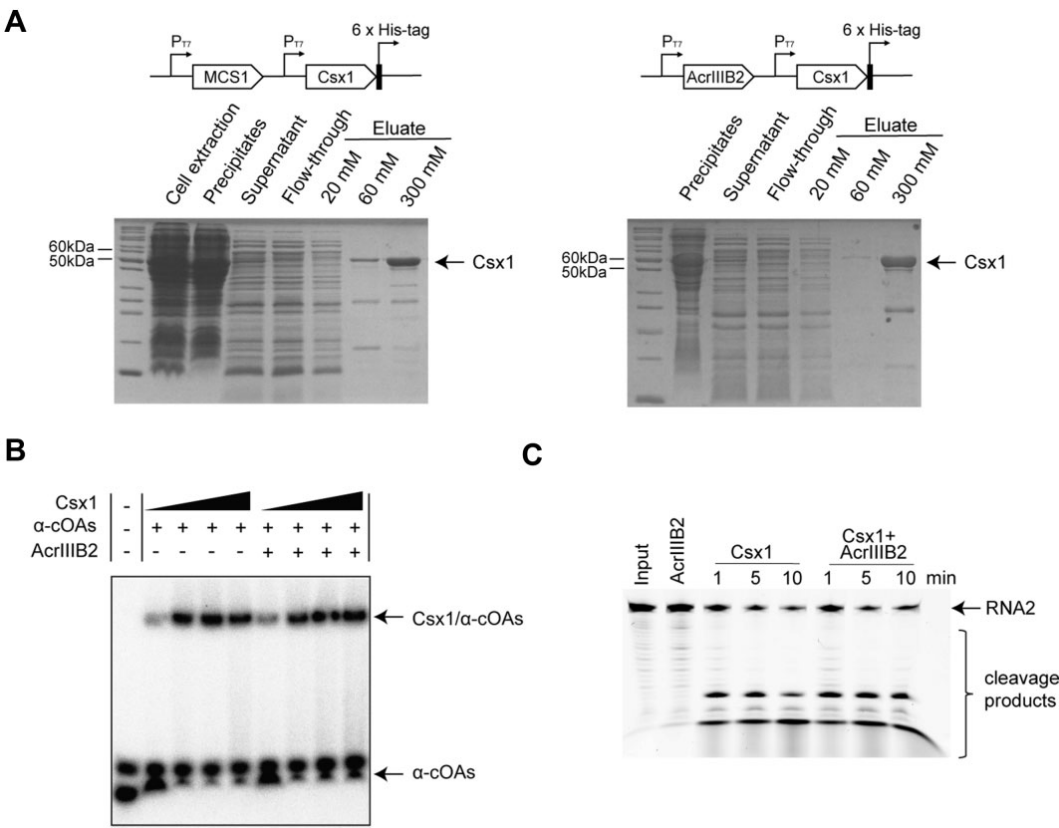
AcrIIIB2 has no direct effect on Csx1. (**A**) Co-purification of His-tagged Csx1 and non-tagged AcrIIIB2. Constructs were transformed into *E. coli* BL21(DE3) cells, which were cultured in 500 ml of LB medium containing 25 μg/ml Chloramphenicol at 37°C for 3 h to an OD_600_ of 0.6–0.7, and then induced with 0.1 mM IPTG for 16 h at 12°C. The cells were harvested and resuspended, then lysed by ultrasonication. Supernatant was collected after centrifugation and filtrated with a 0.22-μm filter. The filtered supernatant was loaded onto a Ni-NTA agarose column. The column was washed with 10 volumes of elution buffer (50 mM Tris-HCl pH 8.0, 300 mM NaCl and 20 mM Imidazole) and protein eluted by gradient imidazole solution. Samples collected at each step were analysis by SDS–PAGE. (**B**) Binding of Csx1 and α ^32^ P-cOAs. The mixture of Csx1 (250 nM, 500 nM, 1 μM and 2 μM), AcrIIIB2 (2 μM) and α ^32^ P-cOAs (500 nM) were incubated at 70°C for 5 min. (**C**) AcrIIIB2 could not inhibit Csx1 RNA cleavage activity. Csx1 (50 nM) or Csx1 (50 nM) with AcrIIIB2 (250 nM) were incubated with 1 μM cold cOAs at 70°C for 1, 5 and 10 min. Samples were separated by a 18% denaturing PAGE gel.

The cOA signals synthesized by the Cmr-α complex can activate the collateral RNase activity of Csx1 that is vital for Type III-B immunity against the middle/late-expressed viral genes (28,45). Csx1 would be activated by cOAs and cleaved RNA non-specifically (46), which caused cell death or cell dormancy. However, we did not observe the cell death or cell dormancy phenomenon during our SMV1 infection experiments that suggests to us AcrIIIB2 might prevent this situation. To explore the effect of AcrIIIB2 on Csx1, we first performed the co-expression assay *in vivo*. However, the results showed that AcrIIIB2 could not be eluted with Csx1-His together (Figure 6A). We then investigated the interaction between AcrIIIB2 with Csx1 *in vitro*. MBP-tagged AcrIIIB2 failed to form a complex with Csx1 and both of the two proteins eluted in the same fraction as the solo protein (Supplementary Figure S10A and B). AcrIIIB2-His also failed to co-elute with Csx1-His in our gel filtration assay (Supplementary Figure S10C and D). These results showed that AcrIIIB2 could not interact with Csx1 directly. We then performed EMSA assay to explore the effect of AcrIIIB2 on the binding between Csx1 and cOAs. The results showed that AcrIIIB2 did not bind or degrade cOAs and had no effect on binding between Csx1 and cOAs (Figure 6B and Supplementary Figure S11A). We then performed RNA cleavage assays with Csx1. The addition of AcrIIIB2 did not affect RNA cleavage activity of Csx1 (Figure 6C). We replaced the synthesized cOAs with the cOA synthesis components including Cmr-α, cold target RNA and ATP to imitate *in vivo* immune pathway. However, AcrIIIB2 did not influence Csx1 activity under this condition (Supplementary Figure S11B). We also found RNA cleavage activity was inhibited with more AcrIIIB2 added (Supplementary Figure S11C). However, we got the same inhibition phenomenon when we added the corresponding protein dissolving buffer into the reaction, which suggested that the inhibition of Csx1 was caused by the buffer instead of AcrIIIB2. From these results, we found that AcrIIIB2 had no direct effect on Csx1.

## Discussion

Type III CRISPR–Cas immunity system is the most complex among all the known CRISPR–Cas systems, and this system employs a unique target RNA-dependent mechanism to defend against invading MGEs. The RNPs of the well-studied Csm and Cmr can recognize complementary RNA targets and cleave them through the ruler cleavage pattern (44,47). The crRNA- target RNA binding activates Cas10 HD domain to cleave non-specific ssDNA and Cas10 Palm domain to produce cOAs for activation of Type III-associated ribonucleases (48–52). On the other hand, MGEs could evolved to fight back against Type III CRISPR–Cas immunity system by inhibiting these functions.

Until now, only two anti-CRISPRs (AcrIIIB1 and AcrIII1) have been characterized for Type III systems. These two Acrs inhibit TypeIII CRISPR–Cas immunity system programmed to only target the middle/late genes by interfering with the activity of Type III-associated ribonucleases (27,28). *Sulfolobus islandicus* rod-shaped virus 2 (SIRV2)-encoded AcrIIIB1 binds the Cmr RNPs (Cmr-α and Cmr-γ) to inhibit the type III-B CRISPR–Cas immunity system from targeting middle/late viral genes rather than early genes (28), suggesting that AcrIIIB1 hinders Csx1 RNase-related process. However, how AcrIIIB1 inhibits the ribonuclease activity of Csx1 remains to be further investigated. AcrIII1 from a new family of viral ring nuclease has been reported to degrade the cOA signal, cA4 (27). The cA4 can activate the non-specific RNase activity of Csx1 and Csm6 (20,51–52), thus resulting in either cell death or chronic cellular dormancy, which would be adverse for virus propagation (28,45). Therefore, degradation of cA4 by AcrIII1 allows the viruses to evade cleavage by the type III CRISPR–Cas-associated Csx1 and Csm6 that were activated by cA4 (27). However, whether other covered Acrs exist to inhibit *cis* RNase, *trans* ssDNase and the collateral RNase activities of Type III immunity that targets both early and middle/late genes remains unknown.

**Figure 7.**
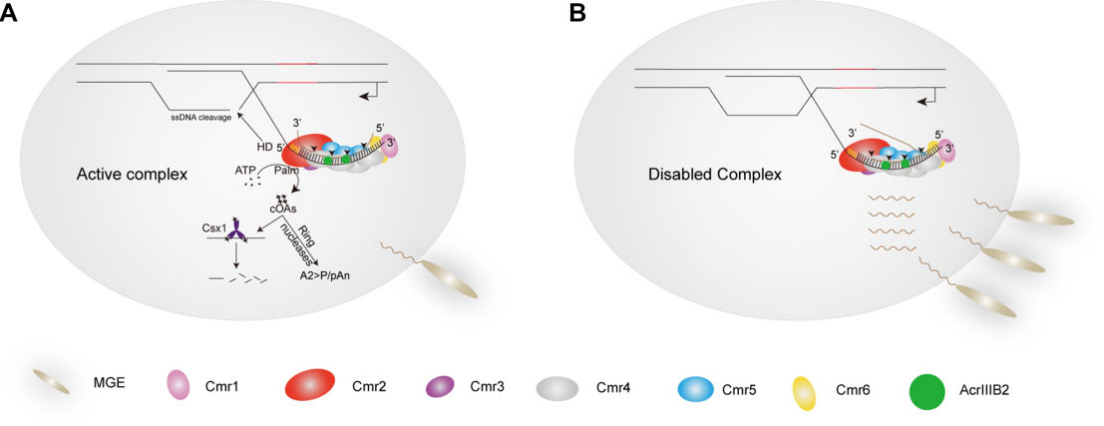
A model for the inhibitory effects of AcrIIIB2 on Type III-B CRISPR–Cas system. (**A**) When virus expresses AcrIIIB2, once the target RNA is cleaved by Cmr-α RNP, AcrIIIB2 binds with intermediate Cmr4α and probably grabs the cleaved target RNA fragments, inhibiting the dissociation of the fragments. Cmr-α is allosterically activated at this step. When the amount of target RNA is less than or equal to that of Cmr-α, it can sustain allosteric activation of Cmr-α and viruses will be eliminated. (**B**) When target RNA is more than the amount of Cmr-α, the undisassociated RNA block the access of other target RNA substrates to inhibit Cmr-α activities. As Cmr-α is activated for a short time, a few cOAs are produced and will probably be degraded by host encoded ring nucleases. Under this condition, viruses propagate successfully.

In this study, we identified a virus-encoded novel anti-CRISPR (AcrIIIB2) that inhibits Type III-B CRISPR–Cas immunity regardless of viral early or middle/late gene to be targeted. AcrIIIB2 is the first identified Acr inactivates Type III immunity against viral early expressed gene that could greatly enhance virus survival. We proposed a model (Figure 7) for the inhibitory effects of AcrIIIB2: in the absence of AcrIIIB2, Cmr-α RNP cleaves target viral RNA, leading to allosteric activation of Cmr2α ssDNase activity to eliminate infected mobile genetic elements. Then, cleaved target RNA dissociates from Cmr-α to initiate next cycle of target RNA and ssDNA cleavages. This process continues until the invading MGE is completely eliminated. However, when the virus expresses AcrIIIB2, AcrIIIB2 binds with intermediate Cmr4α and may grab the intermediate cleavage target RNA fragments through its RNA binding ability to inhibit the dissociation of the cleaved fragments and block the cycles of target RNA and ssDNA cleavages. When the amount of target RNA is less than Cmr-α, it can sustain allosteric activation of Cmr-α activities and the viruses will be eliminated (Figure 7A). When the amount of target RNA is more than Cmr-α, continuous cleavage of target RNA will be inhibited. As a result, ssDNA cleavage and cOA synthesis will also be inhibited (Figure 7B).

The cOAs-activated collateral RNase activity of Csm6/Csx1 plays an important role in targeting middle/late-expressed viral genes in Type III system (28,45). However, we find that AcrIIIB2 had no direct effect on Csx1: AcrIIIB2 could not interact with Csx1 directly (Figure 6A and Supplementary Figure S10), had no effect on cOAs and on the binding of Csx1 with cOAs (Figure 6B and Supplementary Figure S11A) and could not inhibit Csx1 RNase activity *in vitro* (Figure 6C and Supplementary Figure S11B-D). However, AcrIIIB2 influences the Csx1 related pathway: cOAs synthesis. Our data showed that AcrIIIB2 inhibits the dissociation of cleaved target RNA from Cmr-α thereby silent Cmr-α to produce less cOAs (Figure 5H). Although synthesis of cOAs was not completely inhibited by AcrIIIB2, cOAs could be degraded by numerous ring nucleases encoded by the host *Sulfolobus islandicus* REY15A (20,53–55).

It is worth noting that the inhibition of Cmr-α turnover by AcrIIIB2 and the detailed mechanism behind the inhibition were first reported by Lin *et al.* in a preprint article (56), and our results are in line with their data. Although AcrIIIB2 is only encoded by *Sulfolobus* virus SIRV3 with its homolog undetected, our finding infers that more unknown Acrs against Type III CRISPR–Cas systems are probably encoded by the mobile genetic elements and even their hosts. Exploring these unknown Acrs will expand our understanding of the competition between CRISPR–Cas and anti-CRISPRs and could also raise the genetic tools to control CRISPR–Cas systems in diverse applications (57), including Type III CRISPR–Cas-based genome editing (58,59) and RNA targeting (60). In summary, our study provides a new insight into the complexity of the prokaryote-virus arm race and establishes a unique model for studying the structural and functional interplays between Acrs and Type III CRISPR–Cas systems.

## Supplementary Material

gkad804_Supplemental_File

## Data Availability

This study did not generate any unique datasets or code. Plasmids, strains and other unique reagents generated in this study are available upon request.
