# Correction to ‘An archaeal virus-encoded anti-CRISPR protein inhibits type III-B immunity by inhibiting Cas RNP complex turnover’

**DOI:** 10.1093/nar/gkad1107

**Published:** 2023-11-11

*Nucleic Acids Research*, 2023;, gkad804, https://doi.org/10.1093/nar/gkad804

In the originally published version of this manuscript, reference to the following pre-print paper was inadvertently omitted:

‘Lin, Jinzhong & Alfastsen, Lauge & Bhoobalan, Yuvaraj & Peng, Xu. (2023). Molecular Basis for Inhibition of Type Iii-B Crispr-Cas by an Archaeal Viral Anti-Crispr Protein. 10.2139/ssrn.4375100’.

This has now been added to the manuscript as reference 56 and subsequent references have been renumbered accordingly.

As a result, the following paragraph has been corrected:

‘Although AcrIIIB2 is only encoded by *Sulfolobus* virus SIRV3 with its homolog undetected, our finding infers that more unknown Acrs against Type III CRISPR–Cas systems are probably encoded by the mobile genetic elements and even their hosts. Exploring these unknown Acrs will expand our understanding of the competition between CRISPR–Cas and anti-CRISPRs and could also raise the genetic tools to control CRISPR–Cas systems in diverse applications (56), including Type III CRISPR–Cas-based genome editing (57,58) and RNA targeting (59). In summary, our study provides a new insight into the complexity of the prokaryote-virus arm race and establishes a unique model for studying the structural and functional interplays between Acrs and Type III CRISPR–Cas systems.’

This paragraph now reads as follows:

‘It is worth noting that the inhibition of Cmr-α turnover by AcrIIIB2 and the detailed mechanism behind the inhibition were first reported by Lin et al. in a preprint article (56), and our results are in line with their data. Although AcrIIIB2 is only encoded by *Sulfolobus* virus SIRV3 with its homolog undetected, our finding infers that more unknown Acrs against Type III CRISPR–Cas systems are probably encoded by the mobile genetic elements and even their hosts. Exploring these unknown Acrs will expand our understanding of the competition between CRISPR–Cas and anti-CRISPRs and could also raise the genetic tools to control CRISPR–Cas systems in diverse applications (57), including Type III CRISPR–Cas-based genome editing (58,59) and RNA targeting (60). In summary, our study provides a new insight into the complexity of the prokaryote-virus arm race and establishes a unique model for studying the structural and functional interplays between Acrs and Type III CRISPR–Cas systems.’

