# Supplementary material for: An archaeal virus-encoded anti-CRISPR protein inhibits type III-B immunity by inhibiting Cas RNP complex turnover
Source: Nucleic Acids Res. 2023 Oct 18;51(21):11783–96. doi: 10.1093/nar/gkad804 (PMC10681719; doi:10.1093/nar/gkad804)
Supplement: gkad804_Supplemental_File [file gkad804_supplemental_file.pdf]

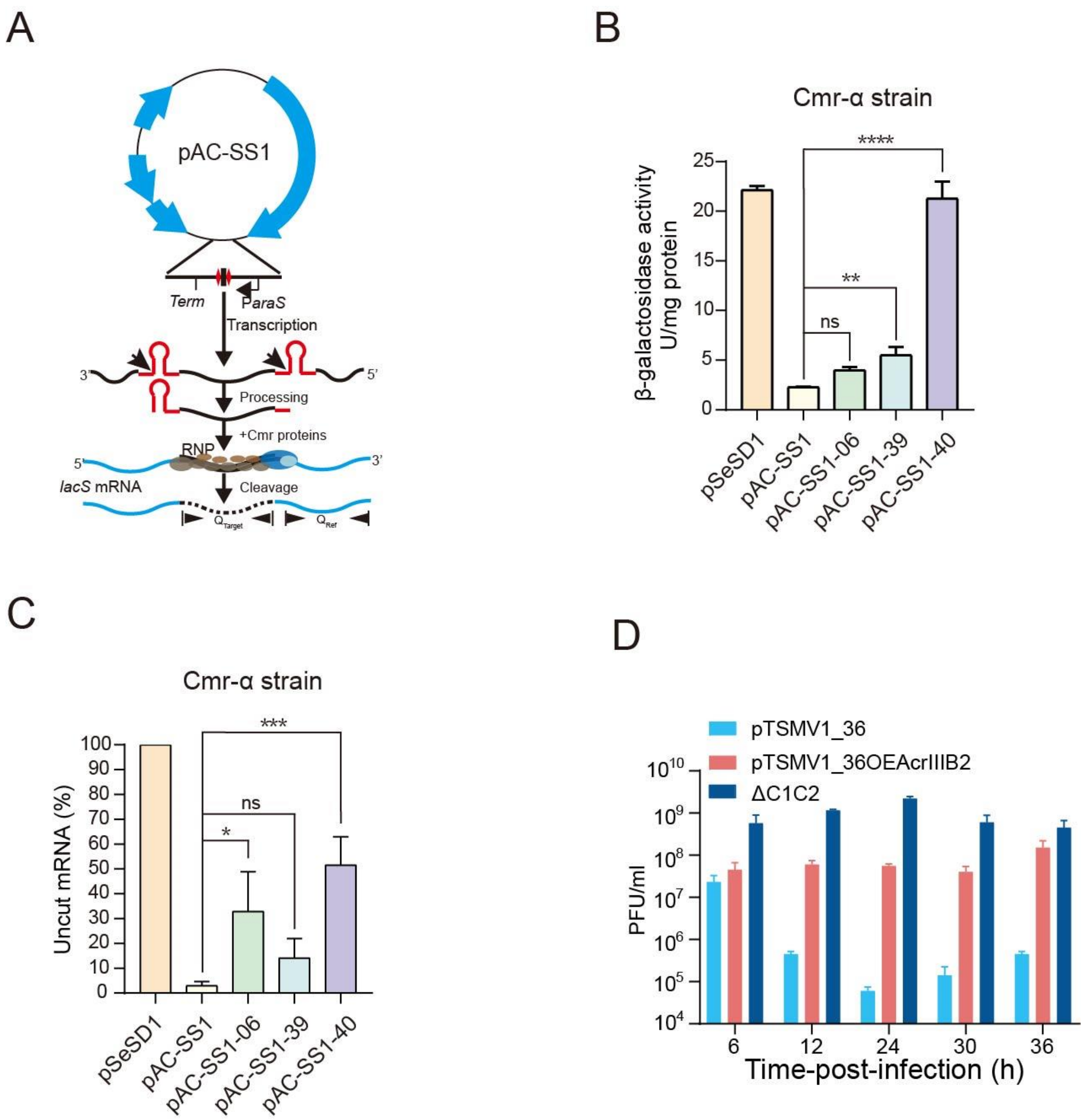

Supplementary Figure 1. AcrIIIB2 inhibits the immunity of Type III-B CRISPR-Cas systems in *S. islandicus.*

A, Schematic of *in vivo* RNA interference activity assay in *S. islandicus.* Plasmid pAC-SS1 was used to produce a crRNA against *lacS* mRNA. Two primer pairs were designed to amplify the target (Q_{Target}) and reference (Q_{Ref}) regions, respectively.

B, Specific β-glycosidase activities in Cmr-α strain carrying empty vector (pSeSD1), RNA interference plasmids pAC-SS1 and pAC-SS1 carrying the expression cassettes for *acr* candidate genes *gp06*, *gp39,* and *gp40*. Cmr-α strain: E233 strain with depletion of I-A and Cmr-β module.

C, mRNA level of uncut *lacS* in Cmr-α strain carrying empty vector (pSeSD1), RNA interference plasmids pAC-SS1 or pAC-SS1 plasmid cloned with the expression cassettes for *acr* candidate genes *gp06*, *gp39,* and *gp40*.

D, Virus titer (plaque-forming units per mL) of SMV1-infected-cultures from (**Fig. 1D**) was measured at 6, 12, 24, 30 and 36 hpi, respectively.

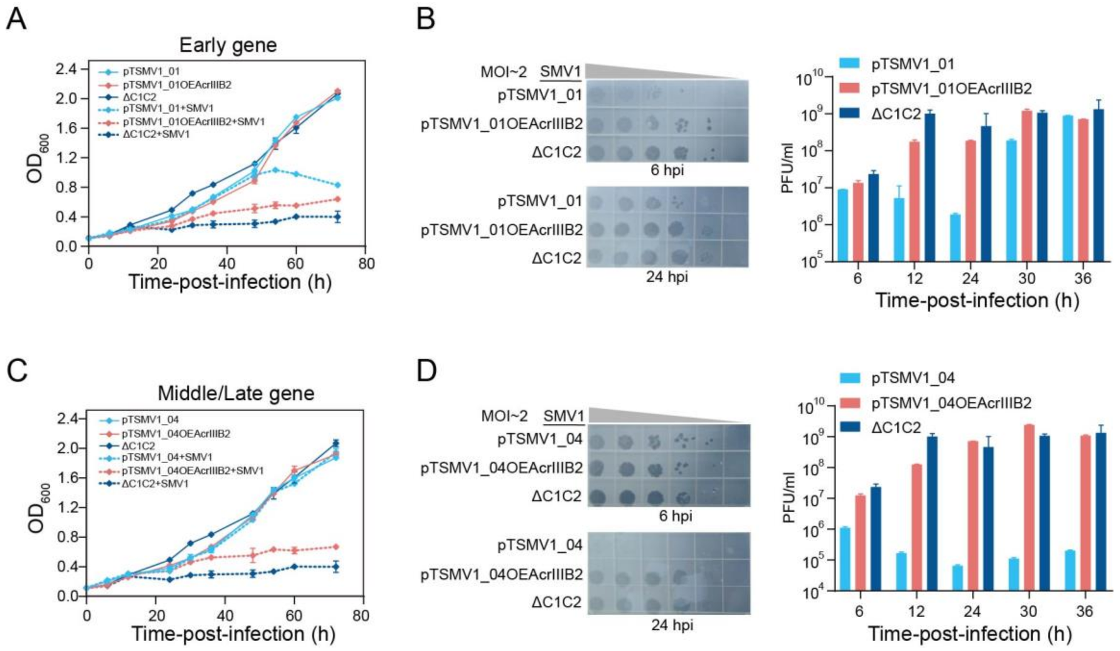

Supplementary Figure 2. AcrIIIB2 inhibits Cmr-α immunity targeting early-expressed or middle/late-expressed viral genes.

A, Growth curve of different cultures with or without SMV1 infection when early-expressed gene *CF87_gp01* was targeted by Cmr-α.

B, Plaques of the supernatant of SMV1-infected-cultures at 6 hpi and 24 hpi from (**A**) on the plates carrying ΔC1C2 cells (left panel) and virus titers of SMV1-infected-cultures from (**A**) was measured at 6, 12, 24, 30 and 36 hpi (right panel).

C, Growth curve of different cultures with or without SMV1 infection when middle/late-expressed gene *CF87_gp04* was targeted by Cmr-α.

D, Plaques of the supernatant of SMV1-infected-cultures at 6 hpi and 24 hpi from (**C**) on the plates carrying ΔC1C2 cells (left panel) and virus titers of SMV1-infected-cultures from (**C**) was measured at 6, 12, 24, 30 and 36 hpi (right panel).

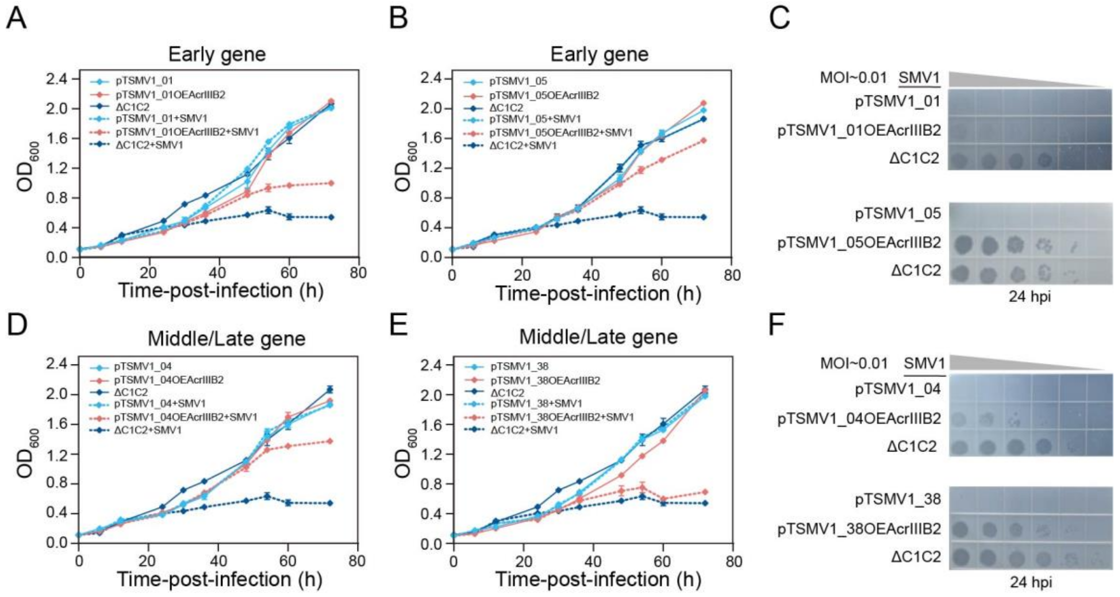

Supplementary Figure 3. AcrIIIB2 inhibits Cmr-α immunity targeting viral early or middle/late genes at MOI~0.01.

Growth curves of ΔC1C2 strain, Cmr-α strain carrying interference plasmid pTSMV1_01 and Cmr-α carrying pTSMV1_01 cloned with AcrIIIB2 expression casstte (**A**) or Cmr-α carrying pTSMV1_05 and Cmr-α carrying pTSMV1_05 cloned with AcrIIIB2 expression casstte (**B**) which were infected with and without SMV1 at MOI~0.01. pTSMV1_01 and pTSMV1_05 target the early expressed genes, *gp01* and *gp05*, respectively.

C, Plaques of 10-fold dilutions of culture supernatants from (**A**) and (**B**) sampled at 24 hpi on the plate spread with ΔC1C2 cells.

D, and **E,** similar to (**A**) and (**B**) in which Cmr-α strain carrying interfernce plasmids against two middle/late genes (*gp04* and *gp38*), respectively, and the interference plasmids expressing AcrIIIB2.

F, similar to (**C**) in which 10-fold dilutions of culture supernatants from (**D**) and (**E**) sampled at 24 hpi were used.

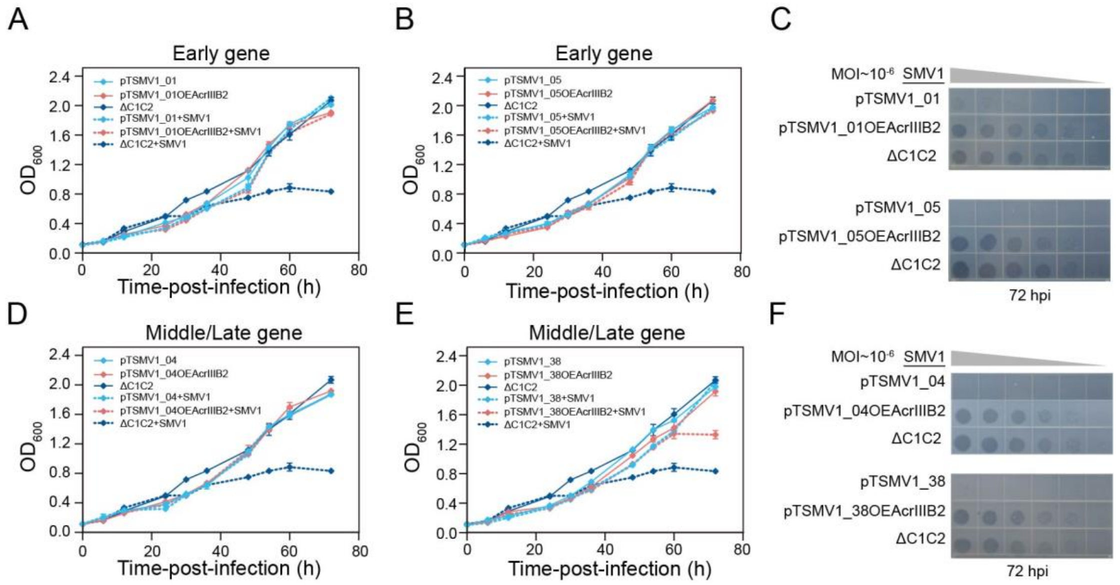

Supplementary Figure 4. AcrIIIB2 inhibits Cmr-α immunity targeting early or middle/late viral genes at MOI~10^{-6}.

Growth curves of ΔC1C2 strain, Cmr-α strain carrying interference plasmid pTSMV1_01 and Cmr-α carrying pTSMV1_01 cloned with AcrIIIB2 expression casstte (**A**) or Cmr-α carrying pTSMV1_05 and Cmr-α carrying pTSMV1_05 cloned with AcrIIIB2 expression casstte (**B**) which were infected with and without SMV1 at MOI~10^{-6}. pTSMV1_01 and pTSMV1_05 target the early expressed genes, *gp01* and *gp05*, respectively.

C, Plaques of 10-fold dilutions of culture supernatants from (**A**) and (**B**) sampled at 72 hpi on the plate spread with ΔC1C2 cells.

D, and **E**, similar to (**A**) and (B) in which Cmr-α strain carrying interfernce plasmids against two middle/late genes (*gp04* and *gp38*), respectively, and the interference plasmids expressing AcrIIIB2.

F, similar to (**C**) in which 10-fold dilutions of culture supernatants from (**D**) and (**E**) sampled at 72 hpi were used.

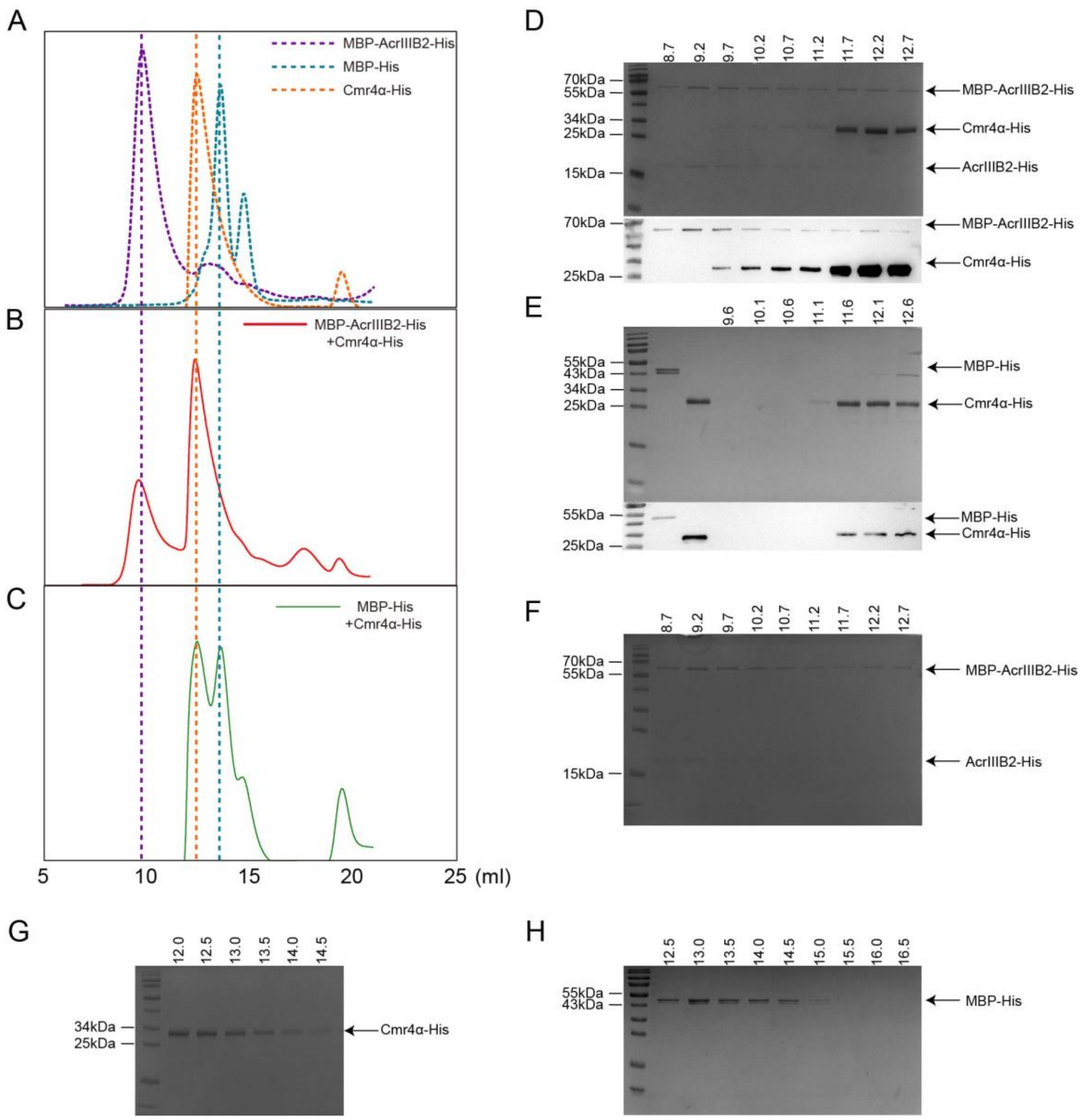

Supplementary Figure 5. AcrIIIB2 interacts with Cmr4α.

A, Gel filtration analysis of the purified MBP-AcrIIIB2-His, MBP-His, and Cmr4α-His.

B, Gel filtration analysis of the purified MBP-AcrIIIB2-His with Cmr4α-His.

C, Gel filtration analysis of the purified MB-His with Cmr4α-His.

D, SDS-PAGE analysis of the gel filtration samples of **(B).**

E, SDS-PAGE analysis of the gel filtration samples of **(C)**.

F~H, SDS-PAGE analysis of the gel filtration samples of the purified MBP-AcrIIIB2-His, Cmr4α-His, and MBP-His individually.

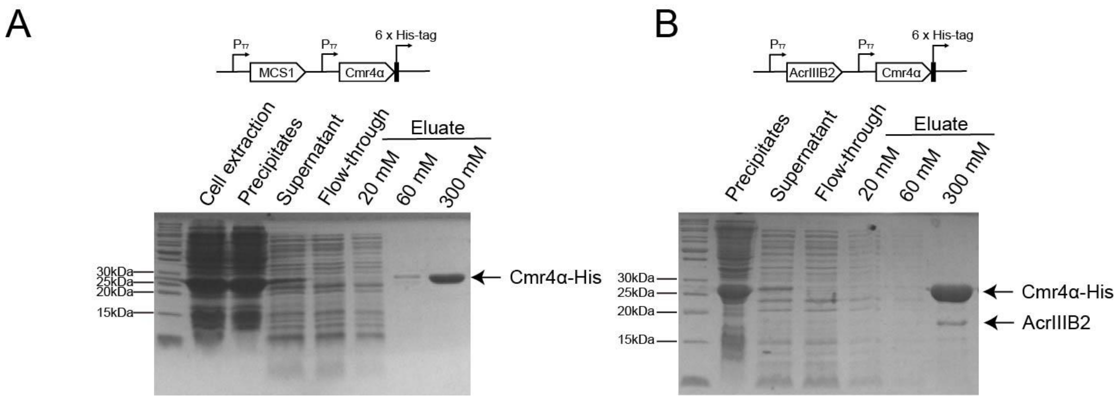

Supplementary Figure 6. AcrIIIB2 interacts with Cmr4α *in vivo*.

A, Purification of Cmr4α-His individually.

B, Co-purification of His-tagged Cmr4α and non-tagged AcrIIIB2 from bacteria expressing Cmr4α and AcrIIIB2 separately under the control of two promoters (as shown on the top of the gel). For (**A)** and (**B)**, plasmids expressing these proteins were transformed into *E. coli* BL21(DE3) cells, which were cultured in 500 ml LB medium containing 25 μg/ml Chloramphenicol at 37°C for 3 h to an OD 600 of 0.6–0.7, and then induced with 0.1 mM IPTG for 16 h at 12°C. The cells were harvested and resuspended, then lysed by ultrasonication. Supernatant was collected after centrifugation and filtrated with 0.22-μm filter. The filtered supernatant was loaded onto a Ni-NTA agarose column. The column was washed with 10 volumes of elution buffer (50 mM Tris-HCl pH 8.0, 300 mM NaCl and 20 mM Imidazole) and protein eluted by gradient imidazole solution. Samples collected at each step were analysis by SDS-PAGE.

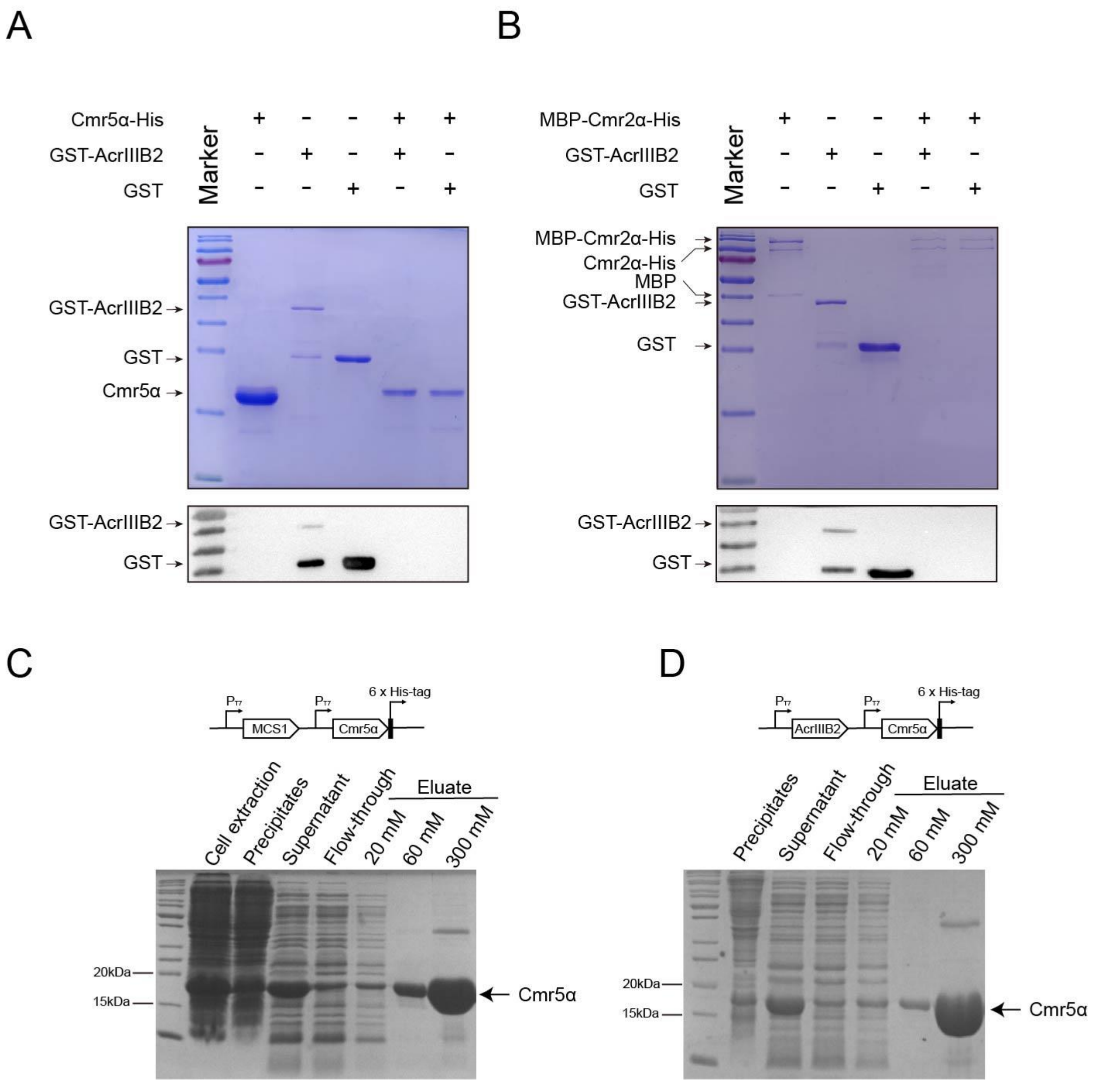

Supplementary Figure 7. AcrIIIB2 shows no interaction with Cmr5α and Cmr2α subunits of *S. islandicus* Type III-B Cmr-α complex.

A, His-tag pull-down assays were performed to detect the interaction between AcrIIIB2 and Cmr5α subunit (top). GST-tagged AcrIIIB2, GST, and Cmr5α-His are indicated by black arrows. Western blot assay was performed to show GST-tagged AcrIIIB2 using the anti-GST antibody (bottom).

B, His-tag pull-down assays was performed to detect the interaction between AcrIIIB2 and Cmr2α subunit (top). GST-tagged AcrIIIB2, GST, and MBP-Cmr2α-His are indicated by black arrows. Western blot assay was performed to show GST-tagged AcrIIIB2 using the anti-GST antibody (bottom).

C, Purification of Cmr5α-His individually.

D, Co-purification of His-tagged Cmr5α and non-tagged AcrIIIB2 from bacteria expressing Cmr5α and AcrIIIB2 separately under the control of two promoters (as shown on the top of the gel). For **(C)** and **(D)**, Constructs were transformed into *E. coli* BL21(DE3) cells, which were cultured in 500 ml LB medium containing 25 μg/ml Chloramphenicol at 37°C for 3 h to an OD 600 of 0.6–0.7, and then induced with 0.1 mM IPTG for 16 h at 12°C. The cells were harvested and resuspended, then lysed by ultrasonication. Supernatant was collected after centrifugation and filtrated

with 0.22-μm filter. The filtered supernatant was loaded onto a Ni-NTA agarose column. The column was washed with 10 volumes of elution buffer (50 mM Tris-HCl pH 8.0, 300 mM NaCl and 20 mM Imidazole) and protein eluted by gradient imidazole solution. Samples collected at each step were analysis by SDS-PAGE.

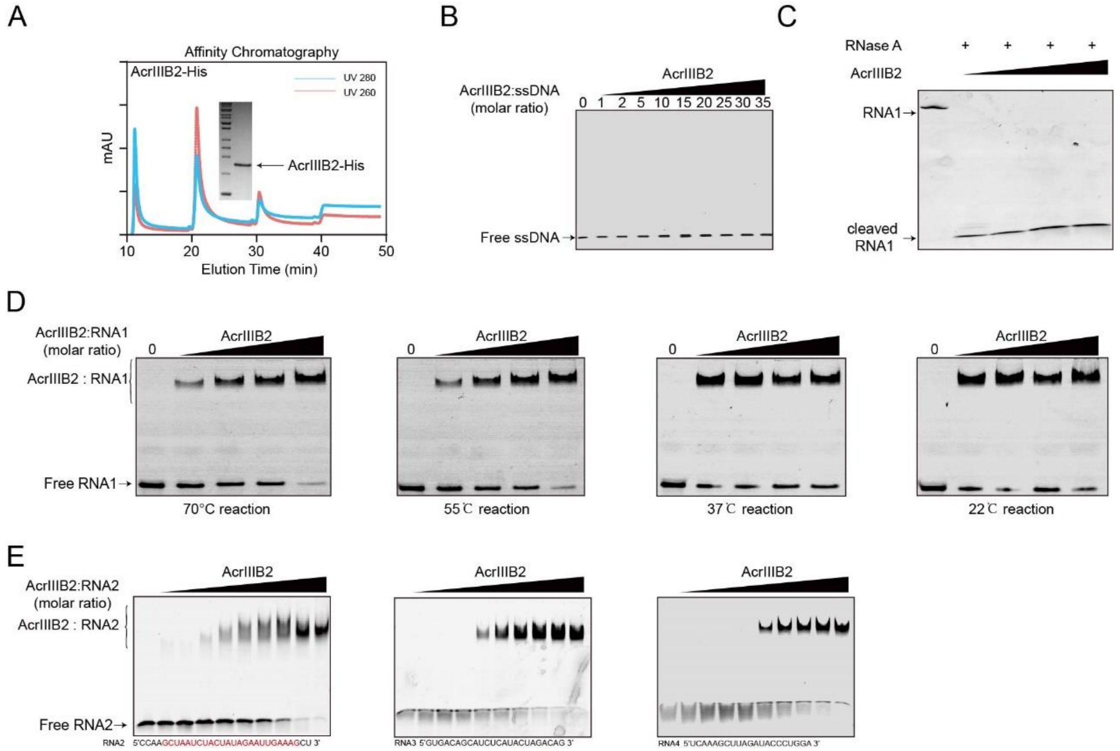

Supplementary Figure 8. AcrIIIB2 non-specifically binds RNA substrates.

A, Affinity chromatography profiles and SDS-PAGE analysis of 6×His-tagged AcrIIIB2. Inside figure: SDS-PAGE analysis of Ni-NTA beads-purified AcrIIIB2 protein.

B, EMSA analysis of 5'-FAM labeled ssDNA with increasing amount of AcrIIIB2.

C, PAGE analysis of the RNase A cleavage products. RNase A (1 μg), 5'-FAM-labeled RNA1 (250 nM), and AcrIIIB2 (0, 100, 400, 800, 1200 nM) were co-incubated for 5 minutes at 70°C and the mixture was used for PAGE analysis.

D, EMSA analysis of the binding between 5'-FAM labeled RNA 1 with AcrIIIB2 at different reaction temperatures.

E, EMSA analysis of the binding between AcrIIIB2 with three different RNA substrates: RNA2, RNA3, and RNA4. EMSA analysis of the binding between AcrIIIB2 with three different RNA substrates: RNA2, RNA3, and RNA4.

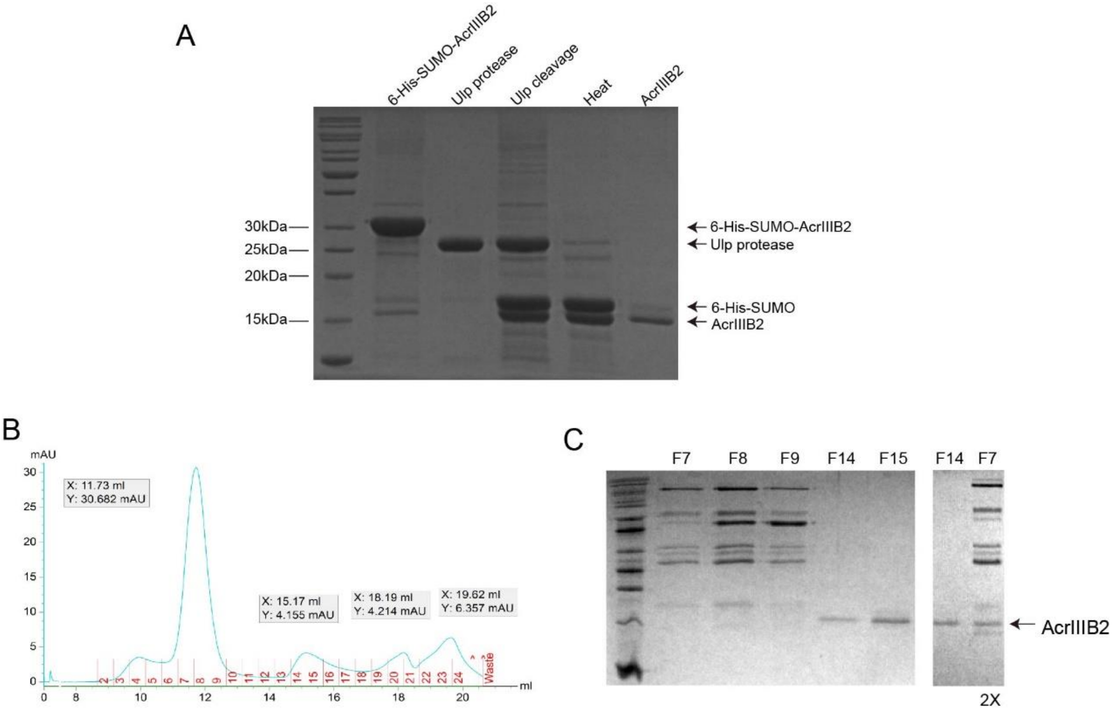

Supplementary Figure 9. Purification of non-tagged AcrIIIB2 and gel filtration analysis of Cmr-α complex with non-tagged AcrIIIB2.

A, The recombinant His-SUMO-AcrIIIB2 protein was expressed in *E. coli* BL21 (DE3) cells carrying plasmid, pET28a-SUMO-AcrIIIB2, induced with IPTG and purified as descripted in Methods. Briefly, the recombinant protein His-SUMO-AcrIIIB2 was digested with SUMO protease at 30 °C for 10 min and heated at 70°C for 20 min (Heat). The sediment was then removed by centrifugation and the supernatant was loaded onto a Ni-NTA column, the flow through fractions containing pure AcrIIIB2 was collected and analyzed by SDS-PAGE.

B, Gel filtration analysis of Cmr-α complex with purified AcrIIIB2 from **(A)**.

C, SDS-PAGE analysis of fraction samples from **(B)**. 2×: sample of this lane is twice more than lane 1 (F7).

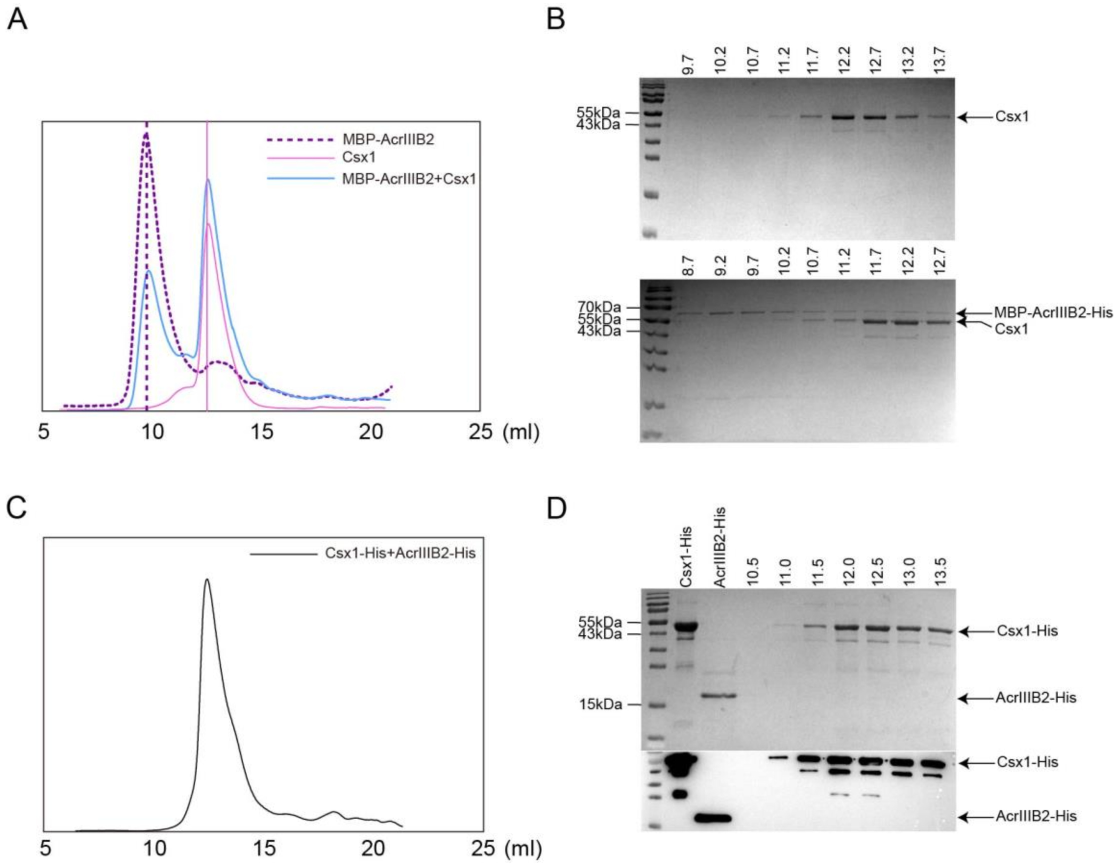

Supplementary Figure 10. AcrIIIB2 does not interact with Csx1.

A, Gel filtration analysis of MBP-AcrIIIB2-His, Csx1-His and mixed MBP-AcrIIIB2-His with Csx1-His.

B, SDS-PAGE analysis of fraction samples from **(A)**.

C, Gel filtration analysis of Csx1-His mixed with AcrIIIB2-His.

D, SDS-PAGE analysis of fraction samples from **(C)**.

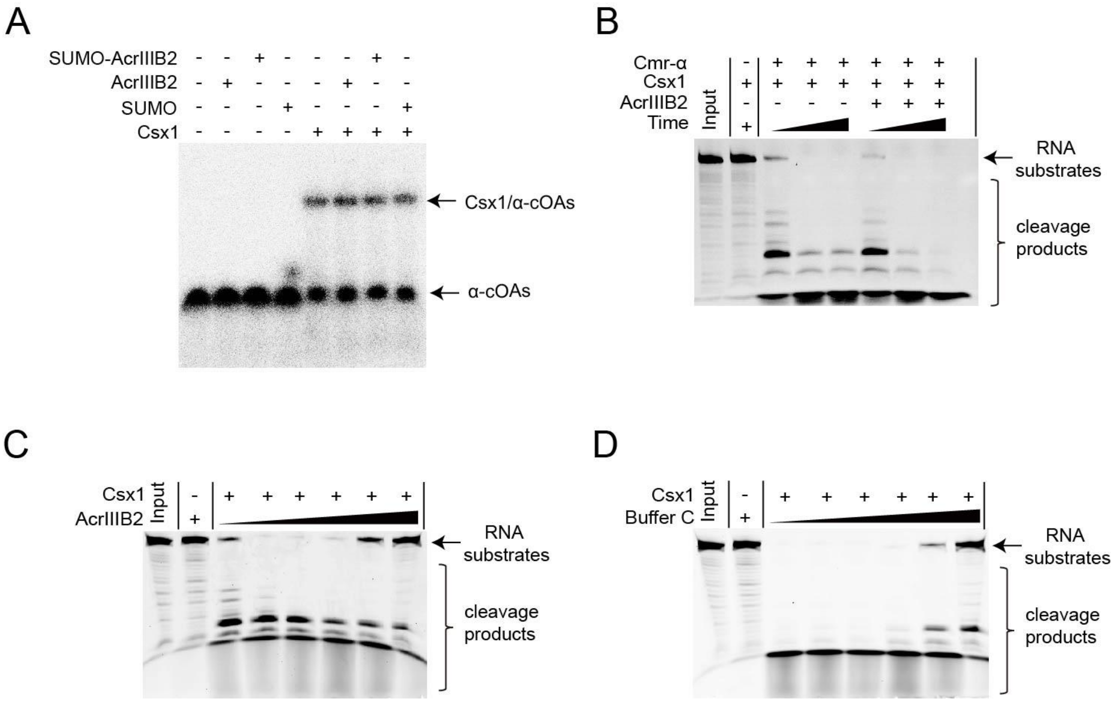

Supplementary Figure 11. AcrIIIB2 does not inhibit Csx1 RNA cleavage activity.
A, Effect of SUMO-AcrIIIB2, AcrIIIB2, SUMO on the binding between Csx1 with α-cOAs individually.
B, Imitation of the Csx1-related immune pathway *in vitro*. In brief, for lane 3 to lane 5, Cmr-α (25 nM), cold target RNA (250 nM), 500 µM ATP, 5' FAM labeled RNA substrates (500 nM) and Csx1 (100 nM) were incubated at 70 °C for 1, 5, 10 min, and then sample were put into ice and added 10 µl 2 X RNA loading (NEB) and incubated at 95 °C for 5 min. For lane 6 to lane 8, Cmr-α-AcrIIIB2 complex (25 nM), cold target RNA (250 nM), 500 µM ATP, 5' FAM labeled RNA substrates (500 nM) and Csx1 (100 nM) were incubated at 70 °C for 1, 5, 10 min, and then sample were put into ice and added 10 µl 2 X RNA loading (NEB) and incubated at 95 °C for 5 min. Sampled were analyzed by denaturing PAGE.
C, and D, Effect of different concentrations of AcrIIIB2 and corresponding concentrations of protein buffer on Csx1 cleavage activity individually. In brief, Csx1 (100 nM), 5' FAM labeled RNA substrates (500 nM), 1µM cold cOAs, and AcrIIIB2 (0, 0.1, 0.2, 0.4, 0.8, 1, 1.2 µM) were incubated at 70 °C for 5 min, and then sample were put into ice and added 10 µl 2 X RNA loading (NEB) and incubated at 95 °C for 5 min. Sampled were analyzed by denaturing PAGE. For (**D**), AcrIIIB2 were displaced with corresponding amounts of buffer G (20 Mm HEPES, pH 7.5, 150 mM NaCl and 10% (v/v) glycerol).

Supplementary Table 1. Strains used in this study

Strain	Description	Source
**Basic strains**		
E.coli		
DH5-α	F⁻ φ80*lac*ZΔM15 Δ(*lac*ZYA *arg*F)U169 *rec*A1 *end*A1 *hsd*R17(r_K^-, m_K^+) *pho*A *sup*E44 λ⁻*thi*-1 *gyr*A96 *rel*A1	Invitrogen
BL21 (DE3)	F⁻*omp*T *hsd*S_B (r_B^-, m_B^-) *gal dcm* (DE3)	Invitrogen
S.islandicus		
E233	Δ*pyrEF*, a spontaneous deletion mutant isolated from Rey15A	(Deng et al, 2009)
E233S1	Δ*pyrEF*Δ*lacS*	(Deng et al, 2009)
Cmr-α	Derived from E233, carrying deletion of III-B Cmr-β locus and I-A locus except Cas6	Lab Strain
ΔC1C2	Derived from S.islandicus REY15A, carrying deletion of both CRISPR locus1 and locus2	(Gudbergsdottir et al., 2011)
E233S1 :: Cmr6α-10His	Derived from E233S1, both carrying an artificial CRISPR locus with 10 copies of spacer and expressing His-tagged Cmr6	(Han et al, 2017)
**E233-derived strains**		
E :: pSeSD	E233 carrying the plasmid pSeSD	(Deng et al, 2009)
E :: pAC-SS1	E233 carrying the plasmid pAC-SS1	This work
E :: pAC-SS1-06	E233 carrying the plasmid pAC-SS1-06	This work
E :: pAC-SS1-39	E233 carrying the plasmid pAC-SS1-39	This work
E :: pAC-SS1-40	E233 carrying the plasmid pAC-SS1-40	This work
E :: pTSMV1_36	E233 carrying the plasmid pTSMV1_36	This work
E :: pTSMV1_36 OEAcrIIIB2	E233 carrying the plasmid pTSMV1_36 OEAcrIIIB2	This work
**Cmr-α-derived strains**		
α :: pAC-SS1	Cmr-α strain carrying the plasmid pAC-SS1	This work
α :: pAC-SS1-06	Cmr-α strain carrying the plasmid pAC-SS1-06	This work
α :: pAC-SS1-39	Cmr-α strain carrying the plasmid pAC-SS1-39	This work
α :: pAC-SS1-40	Cmr-α strain carrying the plasmid pAC-SS1-40	This work
α :: pTSMV1_01	Cmr-α strain carrying the plasmid pTSMV1_01	This work
α :: pTSMV1_04	Cmr-α strain carrying the plasmid pTSMV1_04	This work
α :: pTSMV1_05	Cmr-α strain carrying the plasmid pTSMV1_05	This work
α :: pTSMV1_38	Cmr-α strain carrying the plasmid pTSMV1_38	This work
α :: pTSMV1_01OEAcrIIIB2	Cmr-α strain carrying the plasmid pTSMV1_01OEAcrIIIB2	This work
α :: pTSMV1_04OEAcrIIIB2	Cmr-α strain carrying the plasmid pTSMV1_04OEAcrIIIB2	This work

α :: pTSMV1_05OEAcrIIIB2	Cmr-α strain carrying the plasmid pTSMV1_05OEAcrIIIB2	This work
α :: pTSMV1_38OEAcrIIIB2	Cmr-α strain carrying the plasmid pTSMV1_38OEAcrIIIB2	This work

Supplementary Table 2. Plasmids used in this study.

Plasmids	Description
**Plasmids used for protein expression in *E.coli***	
pET30a-AcrIIIB2	To express His-tagged AcrIIIB2 (*BHS13_gp40*) in *E.coli*
pET30a-Cmr4α	To express His-tagged Cmr4α (*SIRE_RS04485*) in *E.coli*
pET30a-Cmr5α	To express His-tagged Cmr5α (*SIRE_RS04490*) in *E.coli*
pET30a-Csx1	To express His-tagged Csx1 (*SIRE_RS04455*) in *E.coli*
pGEX-6p-1	To express GST in *E.coli*
pGEX-6p-1-AcrIIIB2	To express GST-tagged AcrIIIB2 in *E.coli*
pMAL-C5X-His	To express His-tagged MBP in *E.coli*
pMAL-C5X-His-Cmr2α	To express MBP-His-tagged Cmr2α (*SIRE_RS04505*) in *E.coli*
pMAL-C5X-His-TEV-AcrIIIB2	To express MBP-TE-tagged AcrIIIB2-His (*BHS13_gp40*) in *E.coli*
pET28a-SUMO-AcrIIIB2	To express His-SUMO-tagged AcrIIIB2 (*BHS13_gp40*) in *E.coli*
pACYC_Duet-1-Cmr4α-His	To express His-tagged Cmr4α (*SIRE_RS04485*) in *E.coli*
pACYC_Duet-1-Cmr5α-His	To express His-tagged Cmr5α (*SIRE_RS04490*) in *E.coli*
pACYC_Duet-1-Csx1-His	To express His-tagged Csx1 (*SIRE_RS04455*) in *E.coli*
pACYC_Duet-1-AcrIIIB2-Cmr4α-His	To co-express non-tagged AcrIIIB2 and His-tagged Cmr4α (*SIRE_RS04485*) in *E.coli*
pACYC_Duet-1-AcrIIIB2-Cmr5α-His	To co-express non-tagged AcrIIIB2 and His-tagged Cmr5α (*SIRE_RS04490*) in *E.coli*
pACYC_Duet-1-AcrIIIB2-Csx1-His	To co-express non-tagged AcrIIIB2 and His-tagged Csx1 (*SIRE_RS04455*) in *E.coli*
**Plasmids used for detecting Acr candidates function in *S. islandicus* Rey15A**	
pSeSD1	A *Sulfolobus-E. coli* shuttle vector carrying an expression cassette controlled under a synthetic strong promoter $P_{araS\text{-}SD}$
pSe-Rp	A cloning vector for constructing mini-CRISPR arrays for Sulfolobus
pAC-SS1	Derived from pSe-Rp, carrying an artificial CRISPR locus with 1 S1 spacer of the *S. islandicus lacS* agene, activating specific RNase activity of type III-B CRISPR-Cas systems in *S. islandicus* Rey15A
pAC-SS1-06	Derived from pAC-SS1, carrying *BHS13_gp06* expression cassette controlled under a synthetic strong promoter $P_{araS\text{-}SD}$
pAC-SS1-39	Derived from pAC-SS1, carrying *BHS13_gp39* expression cassette controlled under a synthetic strong promoter $P_{araS\text{-}SD}$
pAC-SS1-40	Derived from pAC-SS1, carrying *BHS13_gp40* expression cassette controlled under a synthetic strong promoter $P_{araS\text{-}SD}$
**Plasmids used for detecting AcrIIIB2 function protecting SMV1 against type III-B CRISPR immunity**	

pTSMV1_01	Derived from pAC-SS1, replaced the spc1 using a spacer targeting *CF87_gp01* of SMV1 to activate III-B CRISPR immunity
pTSMV1_04	Derived from pAC-SS1, replaced the spc1 using a spacer targeting *CF87_gp04* of SMV1 to activate III-B CRISPR immunity
pTSMV1_05	Derived from pAC-SS1, replaced the spc1 using a spacer targeting *CF87_gp0*5 of SMV1 to activate III-B CRISPR immunity
pTSMV1_36	Derived from pAC-SS1, replaced the spc1 using a spacer targeting *CF87_gp36* of SMV1 to activate III-B CRISPR immunity
pTSMV1_38	Derived from pAC-SS1, replaced the spc1 using a spacer targeting *CF87_gp38* of SMV1 to activate III-B CRISPR immunity
pTSMV1_01OEAcrIIIB2	Derived from pTSMV1_01, carrying *AcrIIIB2* expression cassette controlled under a synthetic strong promoter $P_{araS\text{-}SD}$
pTSMV1_04OEAcrIIIB2	Derived from pTSMV1_04, carrying *AcrIIIB2* expression cassette controlled under a synthetic strong promoter $P_{araS\text{-}SD}$
pTSMV1_05OEAcrIIIB2	Derived from pTSMV1_05, carrying *AcrIIIB2* expression cassette controlled under a synthetic strong promoter $P_{araS\text{-}SD}$
pTSMV1_36OEAcrIIIB2	Derived from pTSMV1_36, carrying *AcrIIIB2* expression cassette controlled under a synthetic strong promoter $P_{araS\text{-}SD}$
pTSMV1_38OEAcrIIIB2	Derived from pTSMV1_38, carrying *AcrIIIB2* expression cassette controlled under a synthetic strong promoter $P_{araS\text{-}SD}$

Supplementary Table 3. Primers used in this study.

Name	Sequence (5'-3')
seqF	AACTGGCGGTACATAGTGGTA
seqR	GGGTAGAAGTGTGTATGAG
gp06-F-NdeI	GGAATTCCATATGATGAAAACGAAAATGAGTAAGAA
gp06-R-NheI	CTAGCTAGCTTACCAAAAAGAGTCTTCATCAA
gp39-F-pSeSD	ATGAGGTGAAGCTCATATGATGAGTTTTTACATAATGTATATTGGT
gp39-R-pSeSD	TCCGGAGACGCGTAGCTAGCTTAACTTTCAAATTGACTTTTTAATTT TTC
gp40-F-NdeI	GGAATTCCATATGATGGAAAAAGCACAAGTC
gp40-R-NheI	CTAGCTAGCTTAACTTGATAACAAATATGCTAATAAGA
qTarget-F	AATTCGCCAGATTCTCAGCCTAC
qTarget-R	CGCTCTTTATTCCATCATATGCC
qRef-F	AATTCTTCATTCCAACCGTTGAC
qRef-R	GCTAACGTATCCCTTTTCGGTCT
16sqF	TCAACGCCTGGAATCTTACC
16sqR	CTCGTTGCCTGACTTAACAG
CF87_gp01-spc-F	AAAGTCCTTGAGTATACTAACCCCACCAAGTTCTTTCTGCAACT
CF87_gp01-spc-R	TAGCAGTTGCAGAAAGAACTTGGTGGGGTTAGTATACTCAAGGA
CF87_gp04-spc-F	AAAGCACAGGTATCGATGTTTCTAATGTCTCTAATCTTATCGCA
CF87_gp04-spc-R	TAGCTGCGATAAGATTAGAGACATTAGAAACATCGATACCTGTG
CF87_gp05-spc-F	AAAGAGGTTCGTTGTACTGTTTAACTCTTCATAACTCTTATTTT
CF87_gp05-spc-R	TAGCAAAATAAGAGTTATGAAGAGTTAAACAGTACAACGAACCT
CF87_gp36-spc-F	AAAGTCCCAAACAACGTGGTCATCTTCATCTAACTCCTGCGTGA
CF87_gp36-spc-R	TAGCTCACGCAGGAGTTAGATGAAGATGACCACGTTGTTTGGGA
CF87_gp38-spc-F	AAAGATGTAGTATAGTTGGGAATATATGTTATGGAGGTATAGTG
CF87_gp38-spc-R	TAGCCACTATACCTCCATAACATATATTCCCAACTATACTACAT
Cmr4α-F-NdeI	GGAATTCCATATGACCAAGAGTTATTTAATCCTAGC
Cmr4α-R-SalI	ACGCGTCGACTGAAATCACCTTTATTCTTACAAGT
Cmr5α-F-pET30a	AAGAAGGAGATATACATATGAGCGAATTTGTTAACTTC
Cmr5α-R-pET30a	TGGTGGTGGTGGTGCTCGAGCTCATATGGTAACGCCTC
Csx1-F-NdeI	GGAATTCCATATGAAATGCCTATTTTACATAGCTG
Csx1-R-XhoI	CCGCTCGAGAGCAGATGACAAGAAATTAACT
gp40-F-NdeI	GGAATTCCATATGGAAAAAGCACAAGTCGAAAA
gp40-R-XhoI	CCGCTCGAGACTTGATAACAAATATGCTAATAAGAC
pGST-gp40-F-BamHI	CGGGATCCATGGAAAAAGCACAAGTC
pGST-gp40-R-SalI	ACGCGTCGACTTAACTTGATAACAAATATGCTAATAAGA
pMBP-gp40-F	aggatttcacatatggaaaacctgtattttcagggcatggaaaaagcacaagtc
pMBP-gp40-F	tttcgttttatttgaagcttttagtggtggtgatgatgatg
pSUMO-gp40-F	gagaacagattggtGGATCCatggaaaaagcacaagtcg
pSUMO-gp40-R	TCGAGTGCGGCCGCAAGCTTttaacttgataacaaatatgctaataagacaaga
pACYC-gp40-F	CTTTAATAAGGAGATATACCATGatggaaaaagcacaagtc
pACYC-gp40-F	CCGAGCTCGAATTCGGATCCttaacttgataacaaatatgctaataagacaa
pACYC- Cmr4α-F	TATAAGAAGGAGATATACATATGaccaagagttatttaatcctagc

pACYC- Cmr4α-R	TTACCAGACTCGAGTTAGTGGTGATGATGGTGATGtgaaatcacctttattcttacaag
pACYC- Cmr5α-F	TATAAGAAGGAGATATACATATGagcgaatttgttaacttcg
pACYC- Cmr5α-R	TTTCTTTACCAGACTCGAGTTAGTGGTGATGATGGTGATGctcatatggtaacgcctcc
pACYC- Csx1-F	TATAAGAAGGAGATATACATATGaaatgcctattttacatagc
pACYC- Csx1-R	TCTTTACCAGACTCGAGTTAGTGGTGATGATGGTGATGagcagatgacaagaaattaac

Supplementary Table 4. DNA and RNA oligonucleotides used in this study.

**RNA oligonucleotides**	Sequence (5’-3’)
RNA1 (5’-FAM)	AGUCUCGUAACCAGUCCAAGAACAGAAACCAG UCAAAUGACCG
RNA2 (5’-FAM)	CCAAGCUAAUCUACUAUAGAAUUGAAAGCU
RNA3 (5’-FAM)	UCAAAGCUUAGAUACCCUGGA
RNA4 (3’-FAM)	GUGACAGCAUCUCAUACUAGACAG
non-target RNA	UUGUACUACACAAAAGUACUG
**DNA oligonucleotides**	Sequence (5’-3’)
ssDNA1	TAACACGACTCACATGGGATGAAGTCAGATCAG GGAACGTAGCAGAGGAA
